# Supplementary figures and images for: Rab12 is a regulator of LRRK2 and its activation by damaged lysosomes
Source: eLife. 2023 Oct 24;12:e87255. doi: 10.7554/eLife.87255 (PMC10708889; doi:10.7554/eLife.87255)

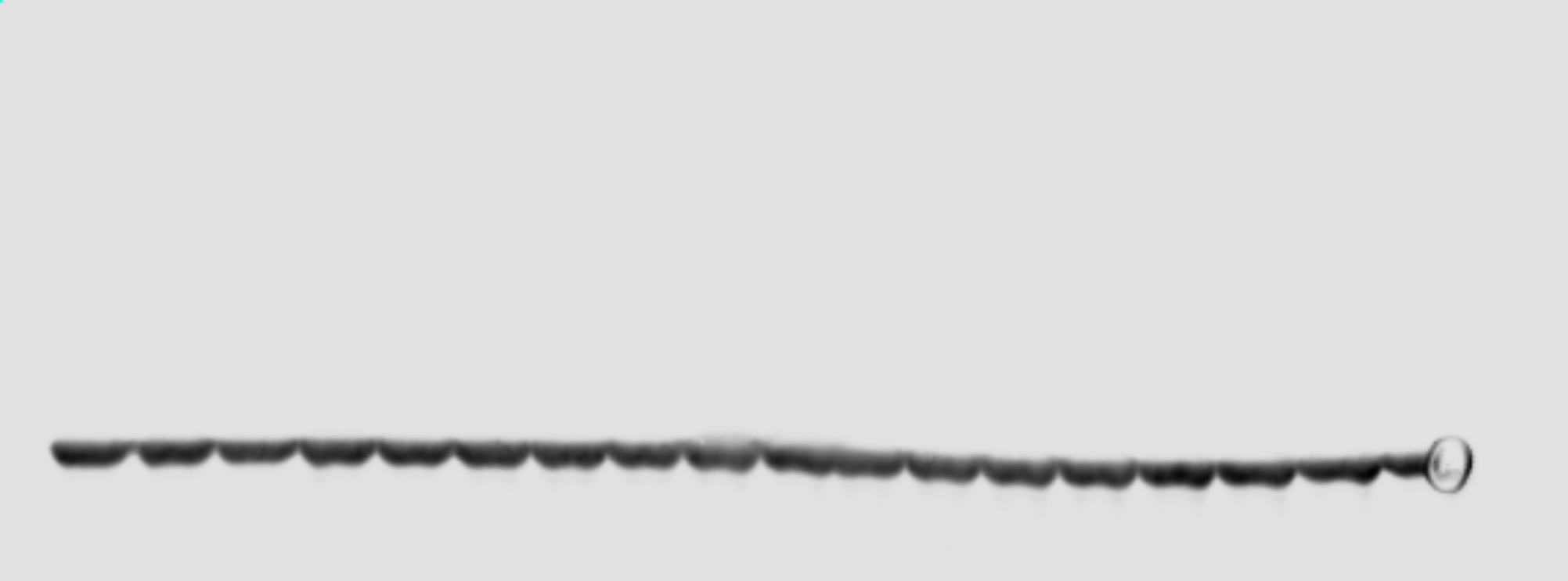

Supplement: Figure 1—source data 1. [file elife-87255-fig1-data1.zip › Figure 1D Source Data GAPDH_800.tif]

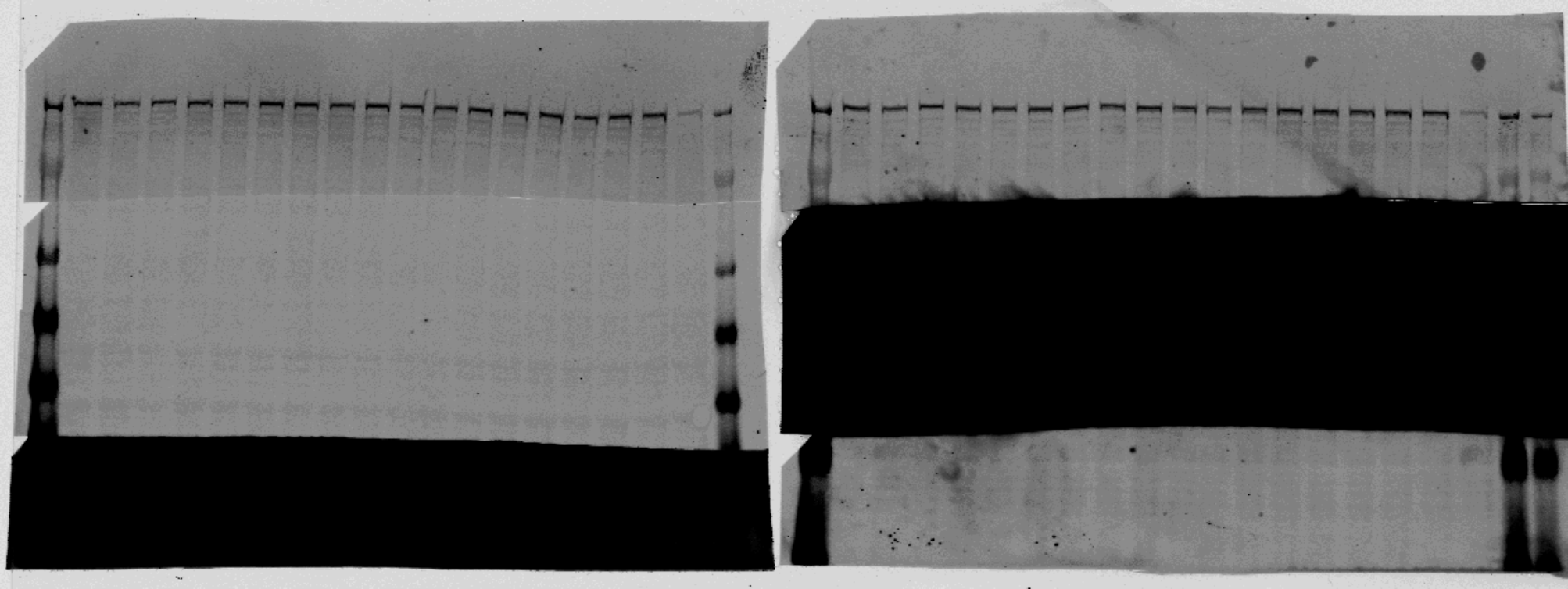

Supplement: Figure 1—source data 1. [file elife-87255-fig1-data1.zip › Figure 1D Source Data LRRK2_800.tif]

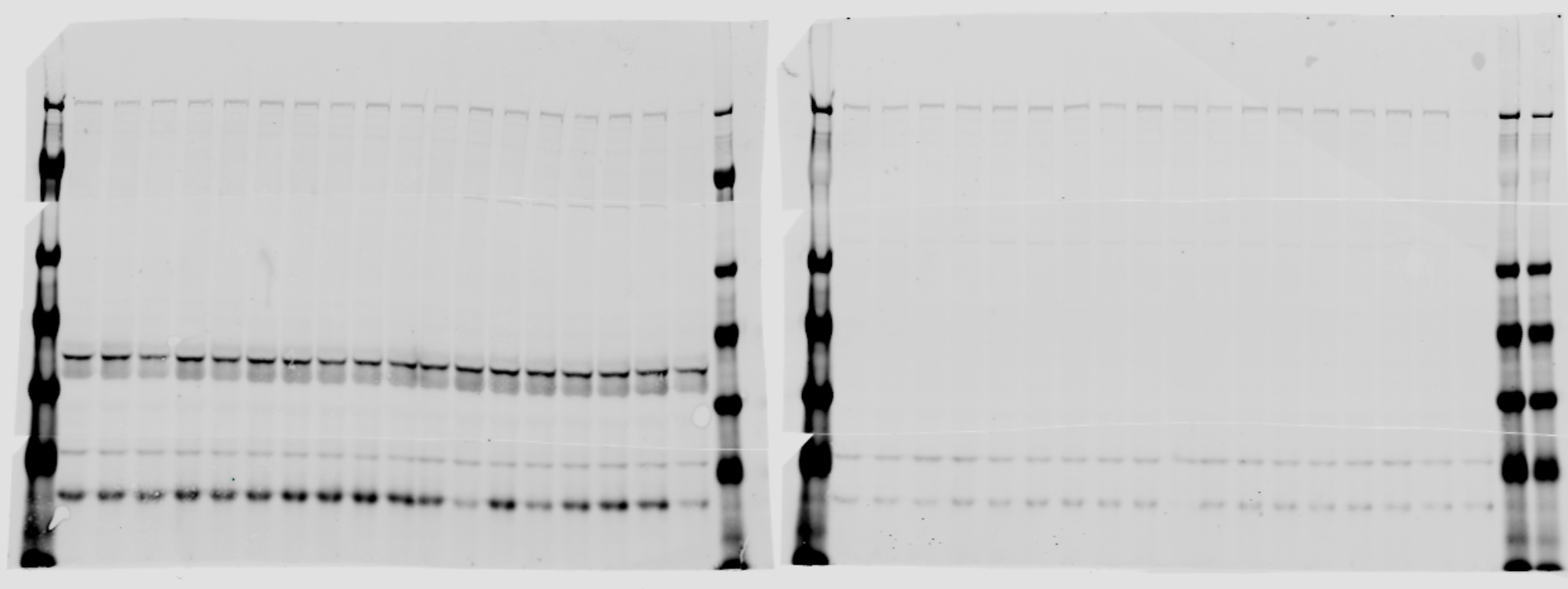

Supplement: Figure 1—source data 1. [file elife-87255-fig1-data1.zip › Figure 1D Source Data pRab10_700.tif]

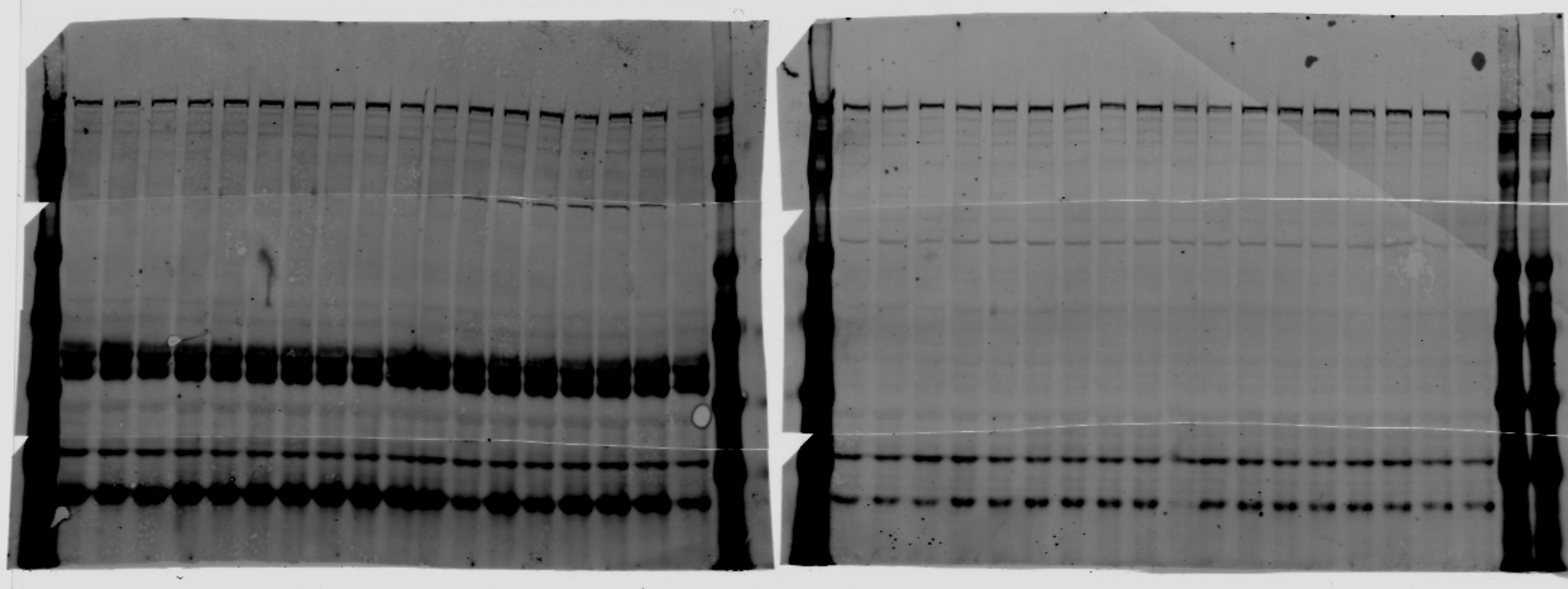

Supplement: Figure 1—source data 1. [file elife-87255-fig1-data1.zip › Figure 1D Source Data Rab8a_700.tif]

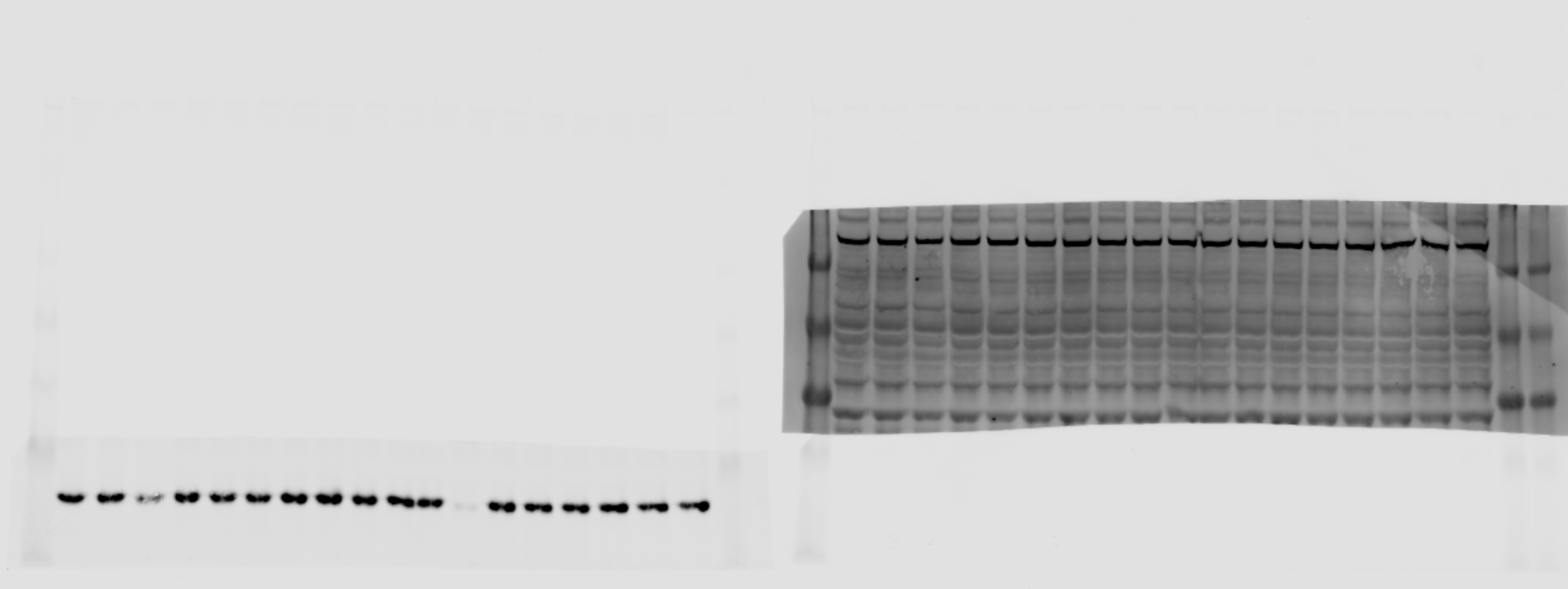

Supplement: Figure 1—source data 1. [file elife-87255-fig1-data1.zip › Figure 1D Source Data Rab10_800.tif]

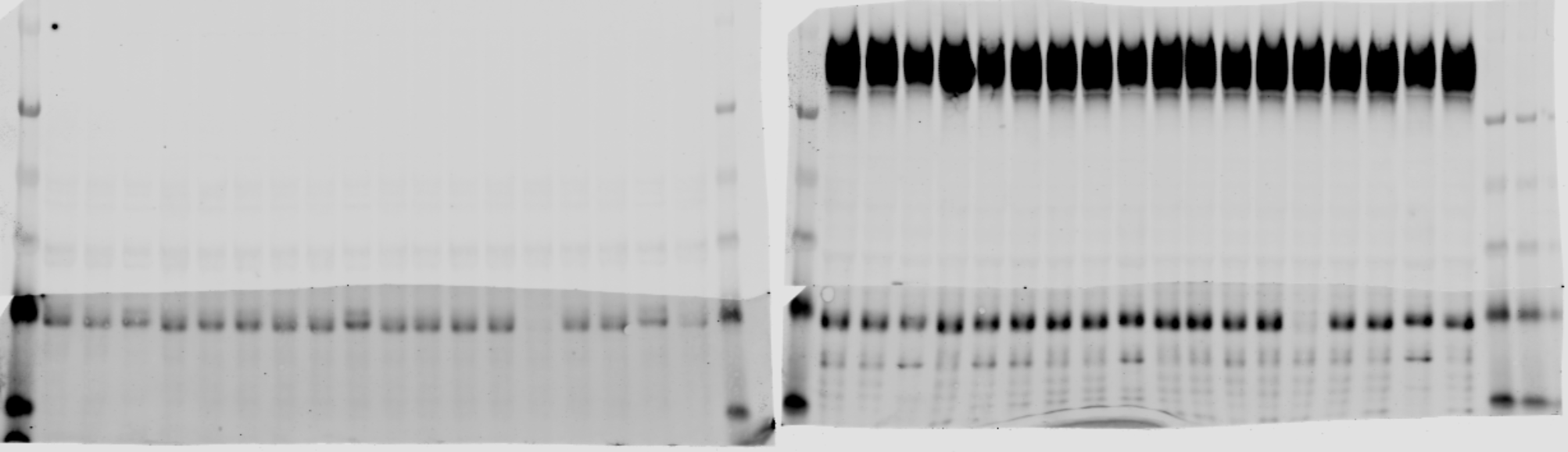

Supplement: Figure 1—source data 1. [file elife-87255-fig1-data1.zip › Figure 1D Source Data Rab12_800.tif]

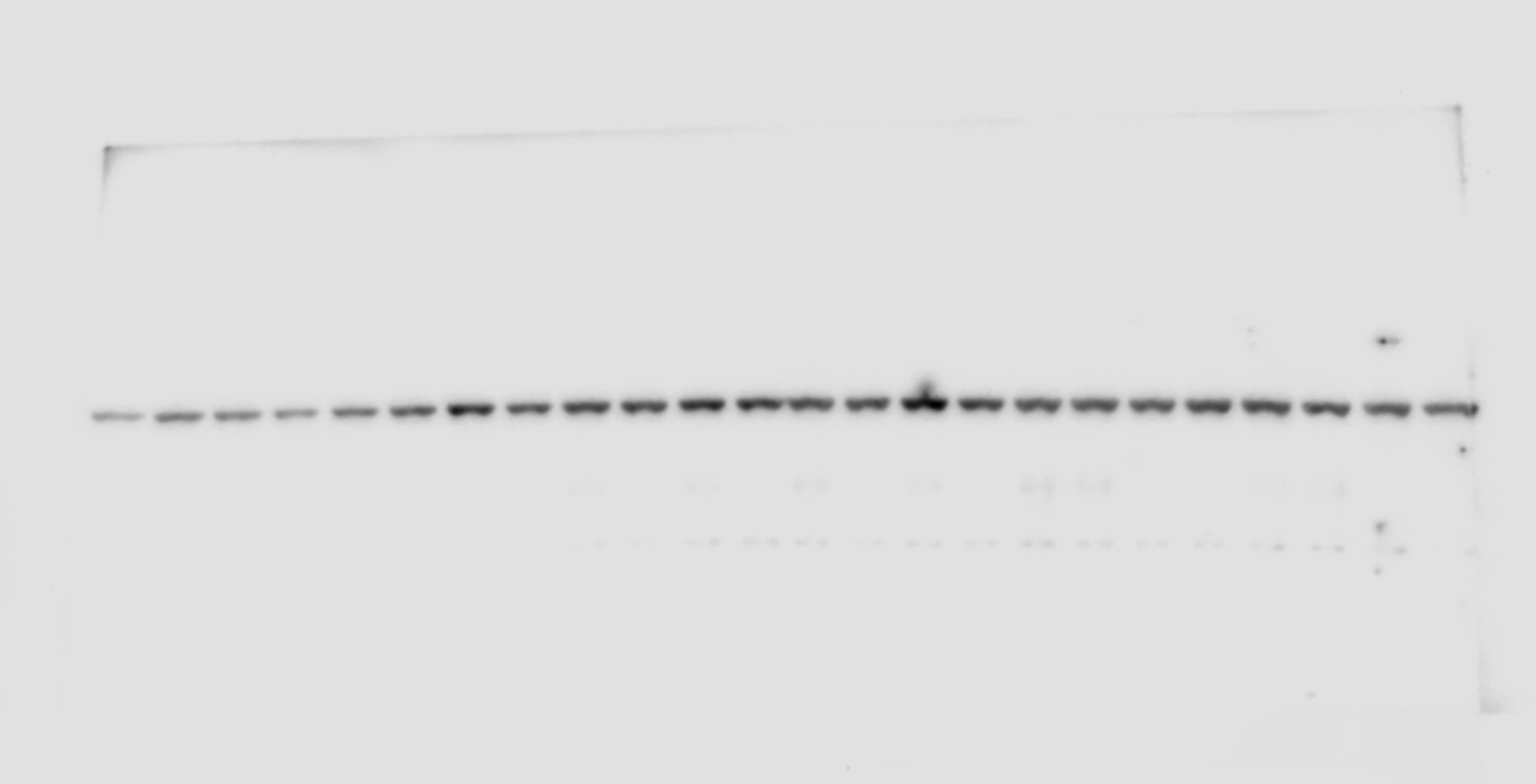

Supplement: Figure 1—source data 1. [file elife-87255-fig1-data1.zip › Figure 1E Source Data GAPDH_chemi.tif]

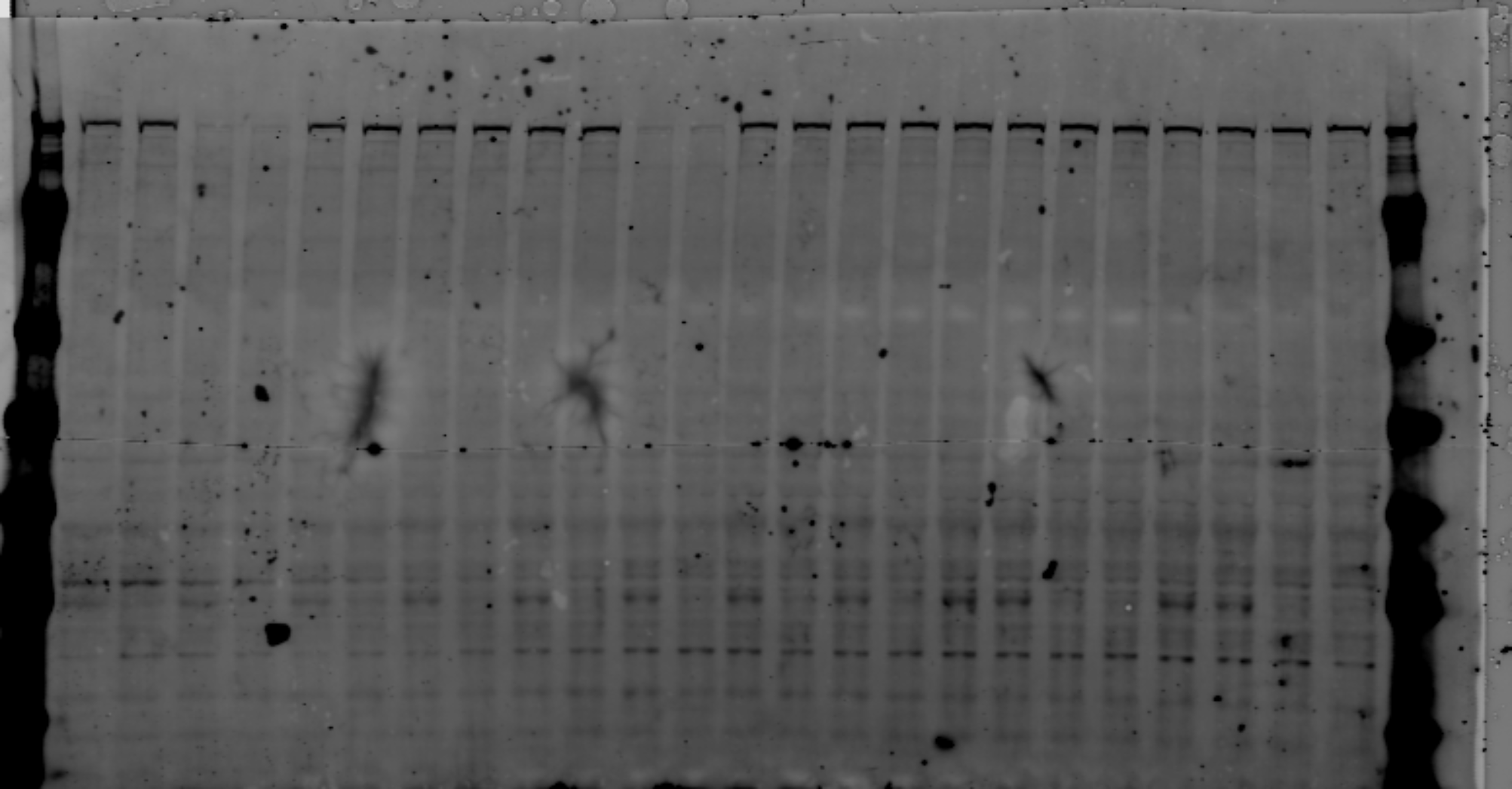

Supplement: Figure 1—source data 1. [file elife-87255-fig1-data1.zip › Figure 1E Source Data Rab12_700.tif]

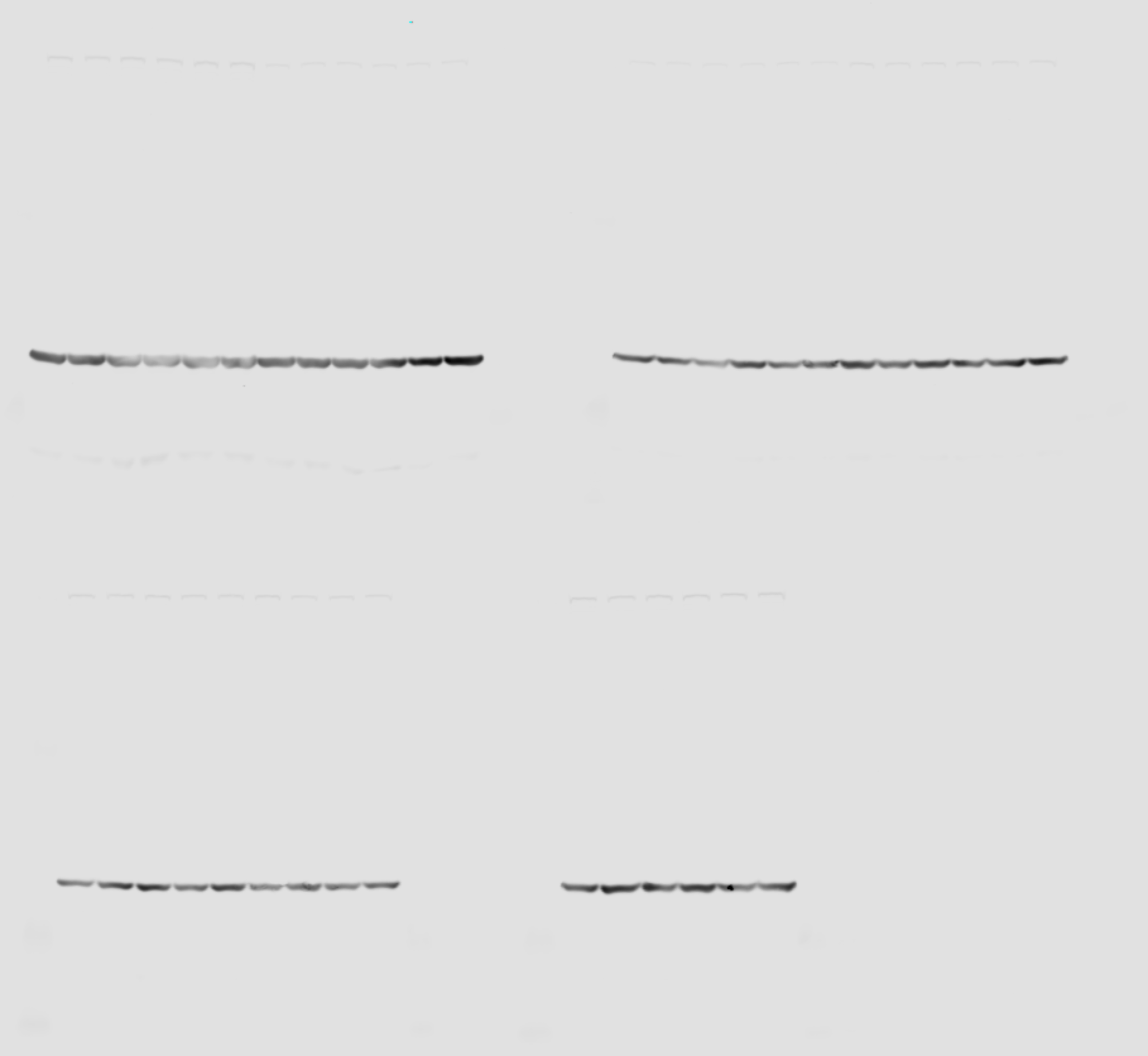

Supplement: Figure 1—source data 1. [file elife-87255-fig1-data1.zip › Figure 1F Source Data Rab10 and GAPDH 1 and 2_800.tif]

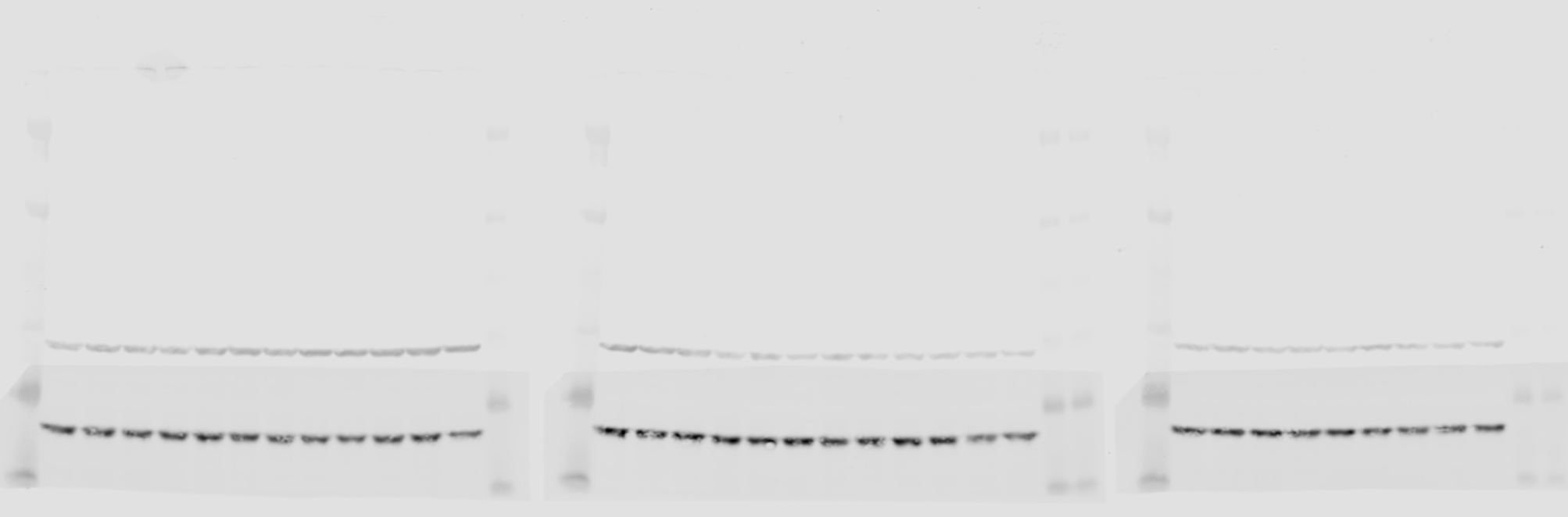

Supplement: Figure 1—source data 1. [file elife-87255-fig1-data1.zip › Figure 1F Source Data Rab10 and GAPDH 3.tif]

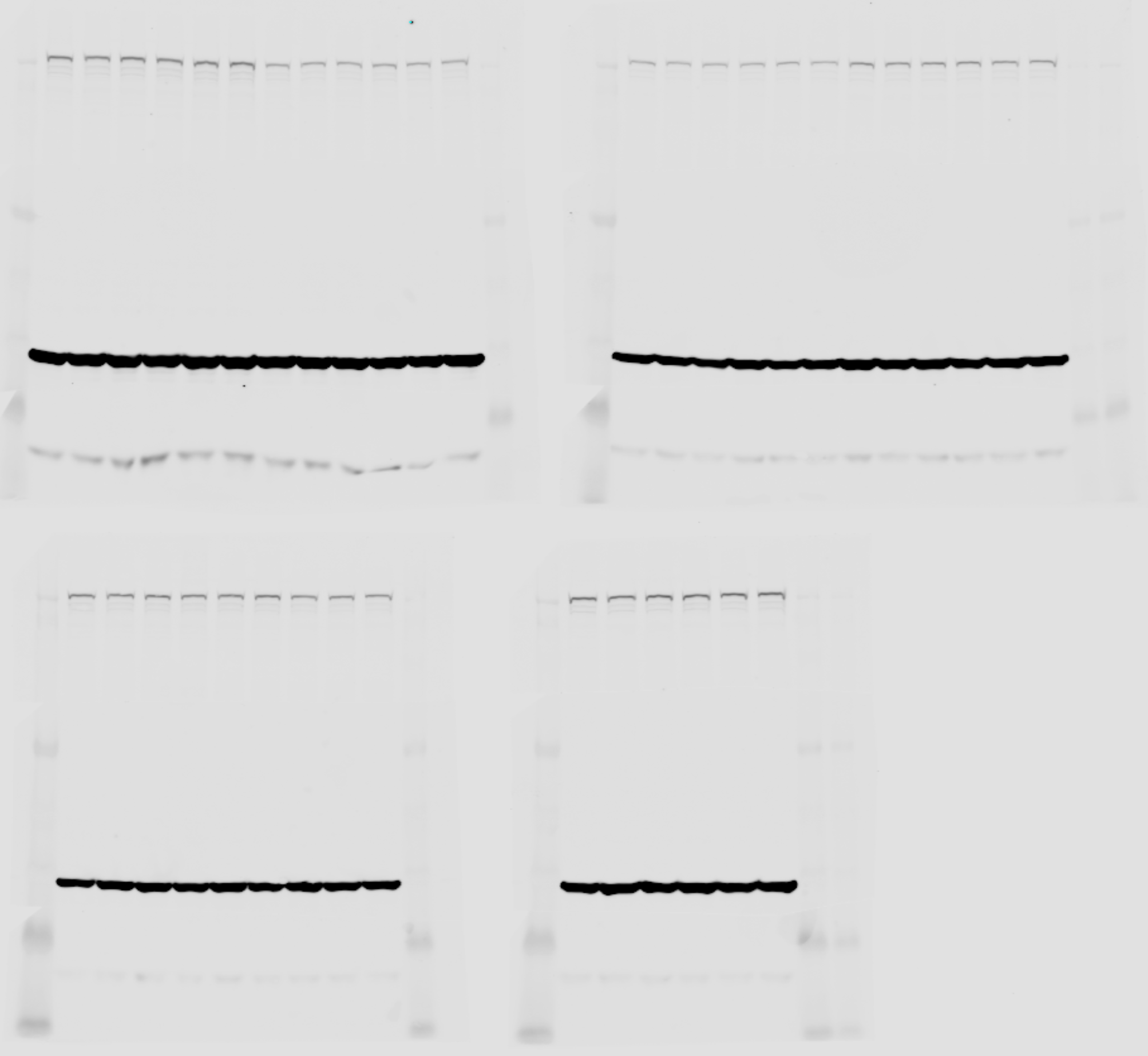

Supplement: Figure 1—figure supplement 1—source data 1. [file elife-87255-fig1-figsupp1-data1.zip › Figure 1F Source Data_LRRK2 Rab10 and GAPDH_800.tif]

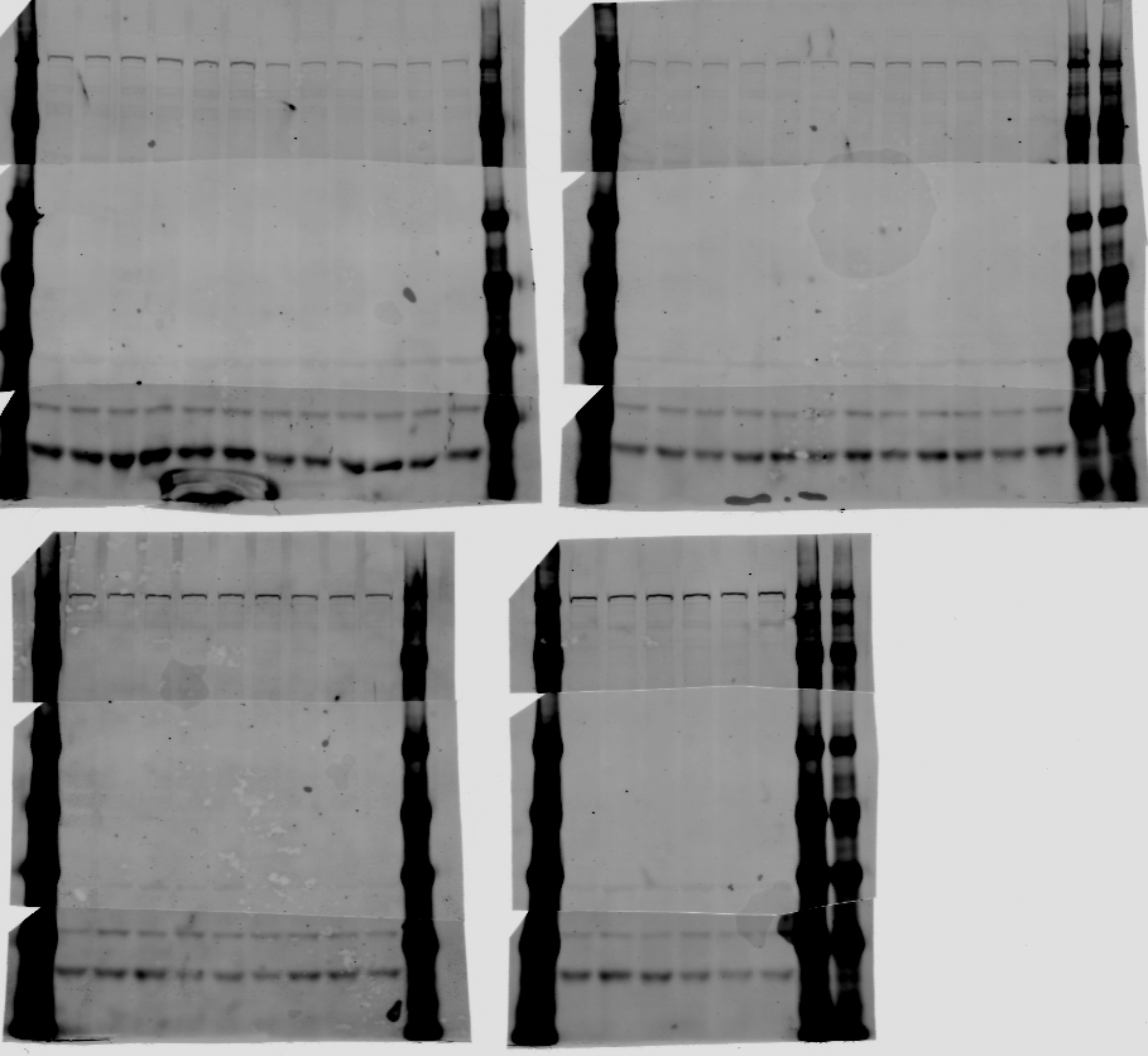

Supplement: Figure 1—figure supplement 1—source data 1. [file elife-87255-fig1-figsupp1-data1.zip › Figure 1F Source Data_pRab10_700.tif]

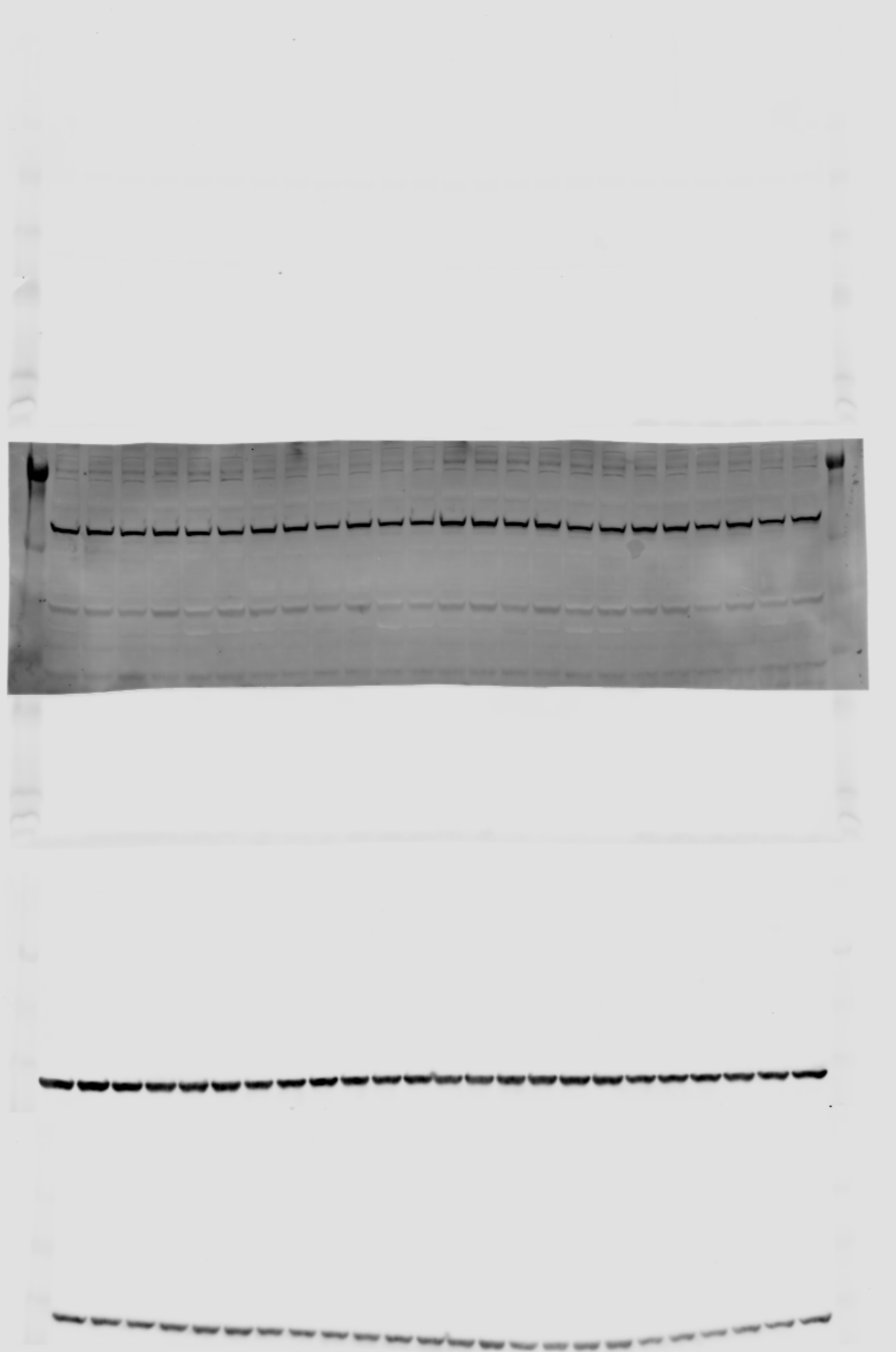

Supplement: Figure 2—source data 1. [file elife-87255-fig2-data1.zip › Figure 2A and C Source Data_GAPDH_800.tif]

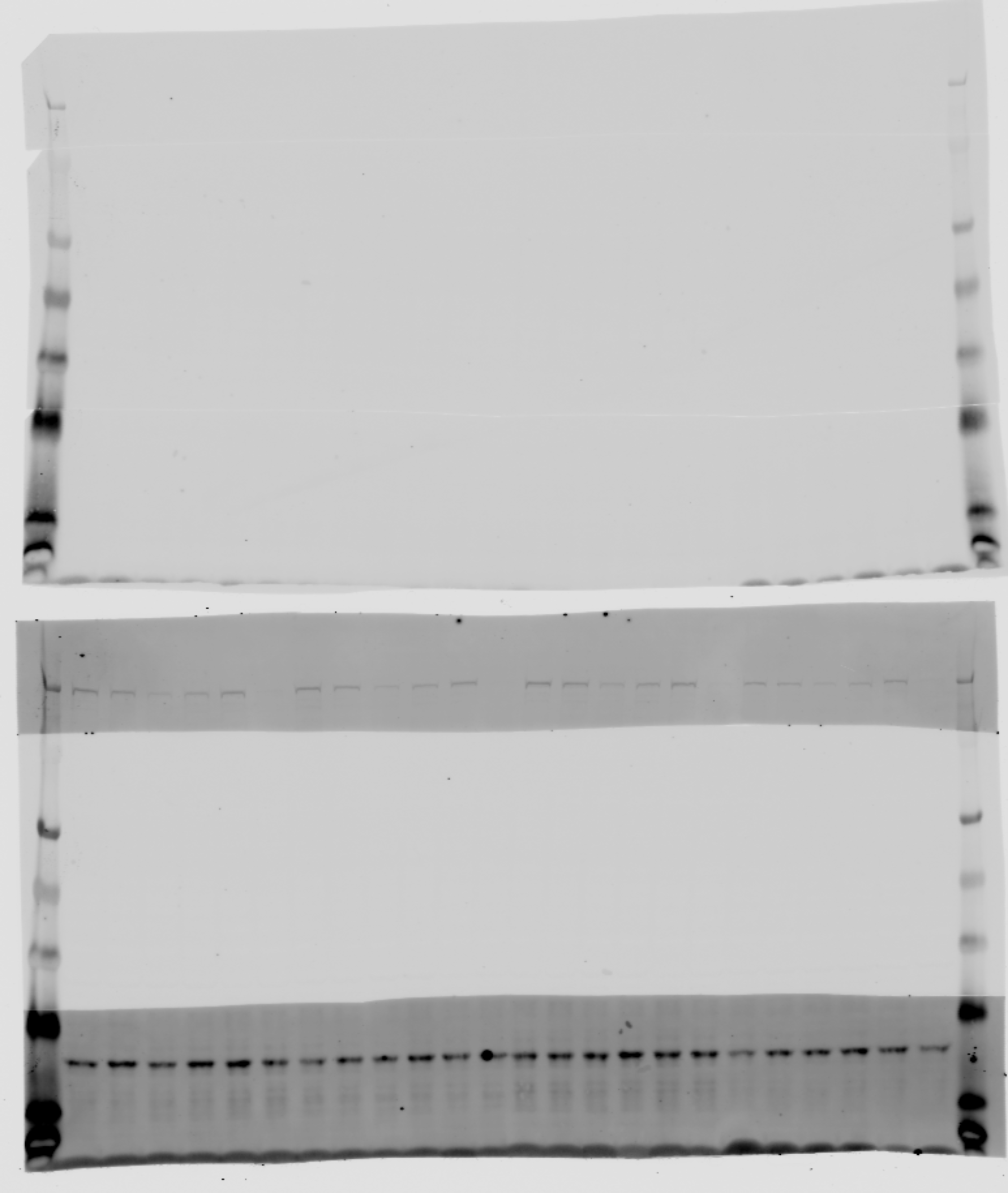

Supplement: Figure 2—source data 1. [file elife-87255-fig2-data1.zip › Figure 2A and C Source Data_Rab10_800.tif]

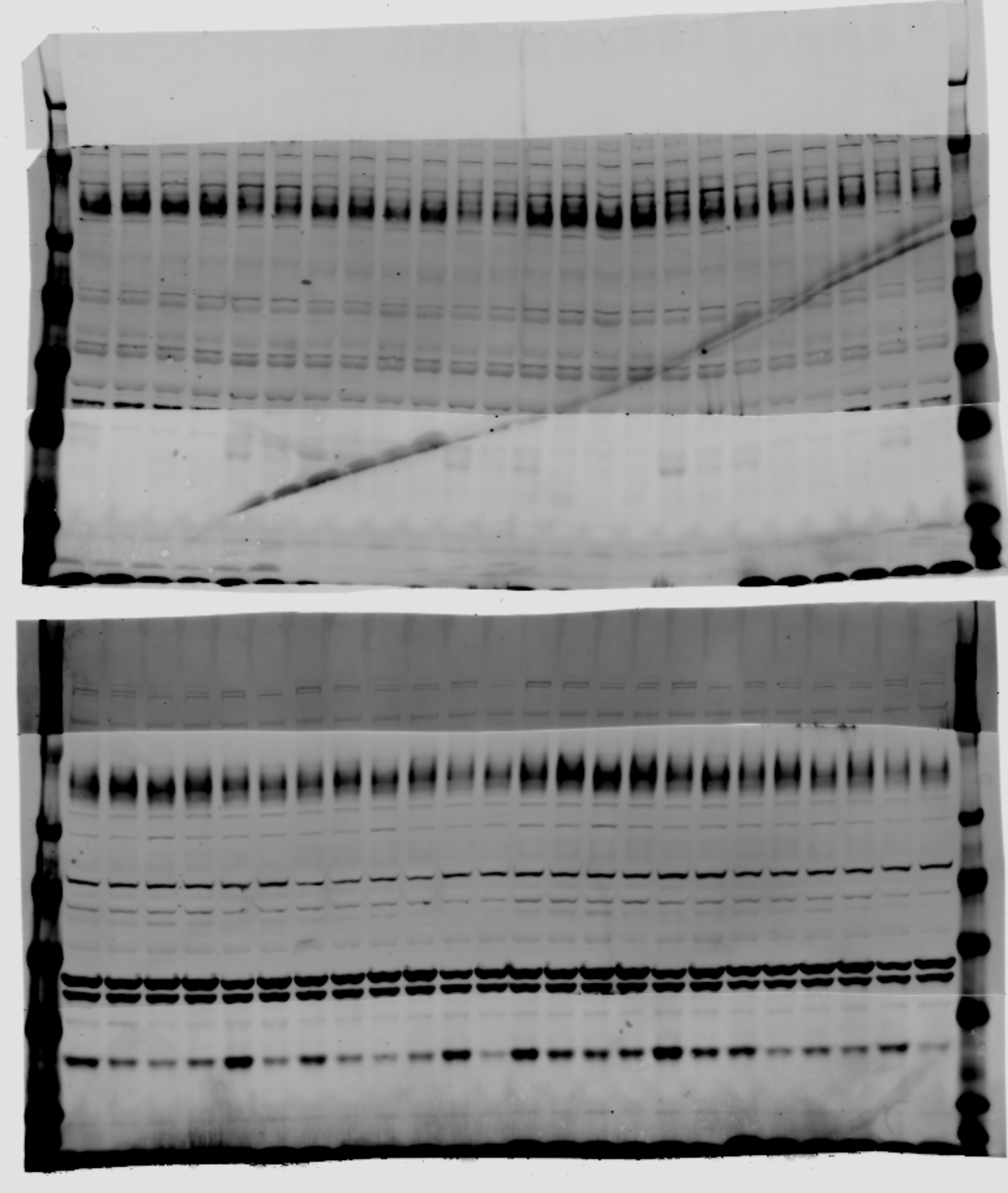

Supplement: Figure 2—source data 1. [file elife-87255-fig2-data1.zip › Figure 2A Source Data_pRab10_700.tif]

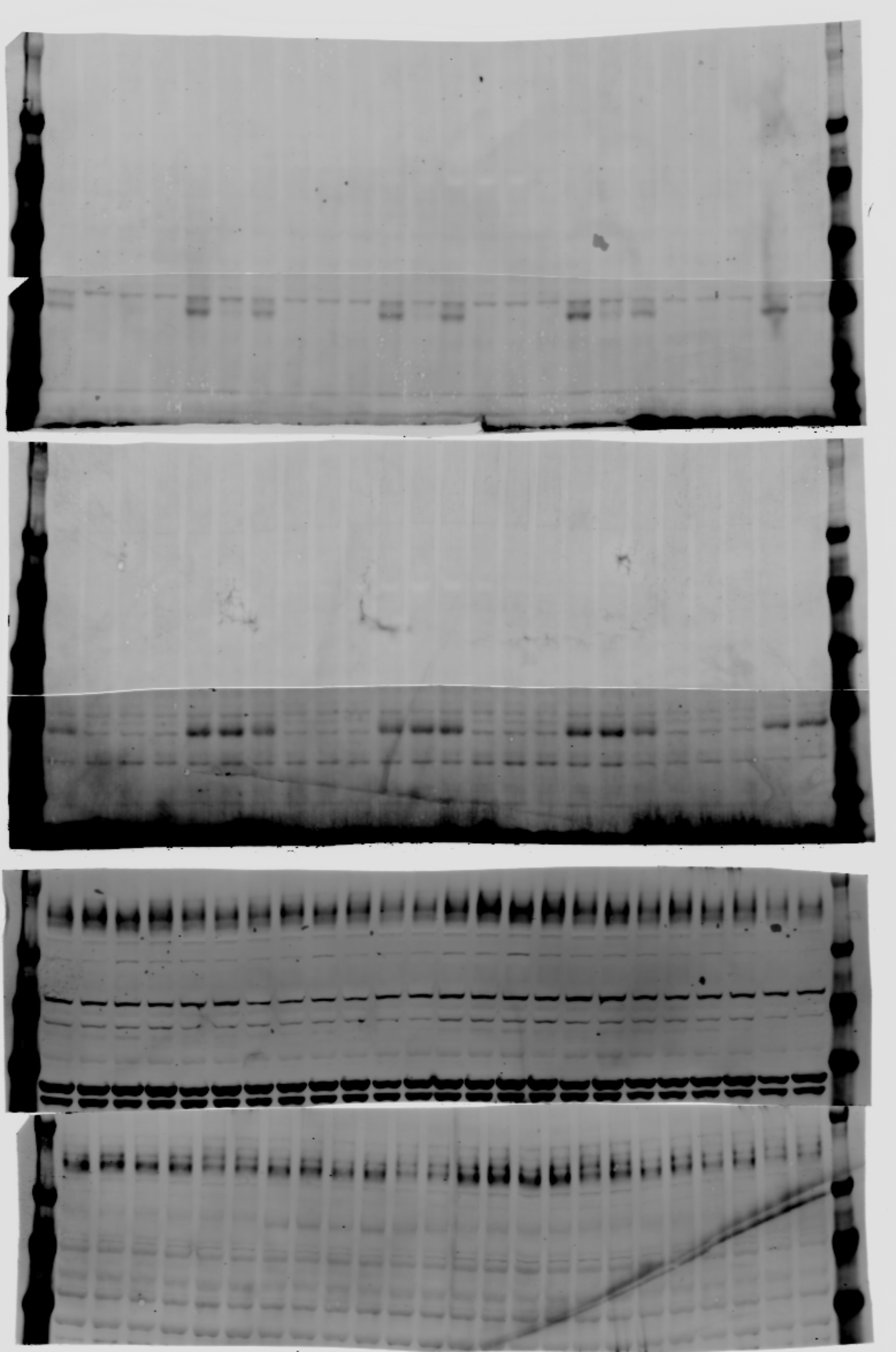

Supplement: Figure 2—source data 1. [file elife-87255-fig2-data1.zip › Figure 2A Source Data_Rab12 and pRab12_700.tif]

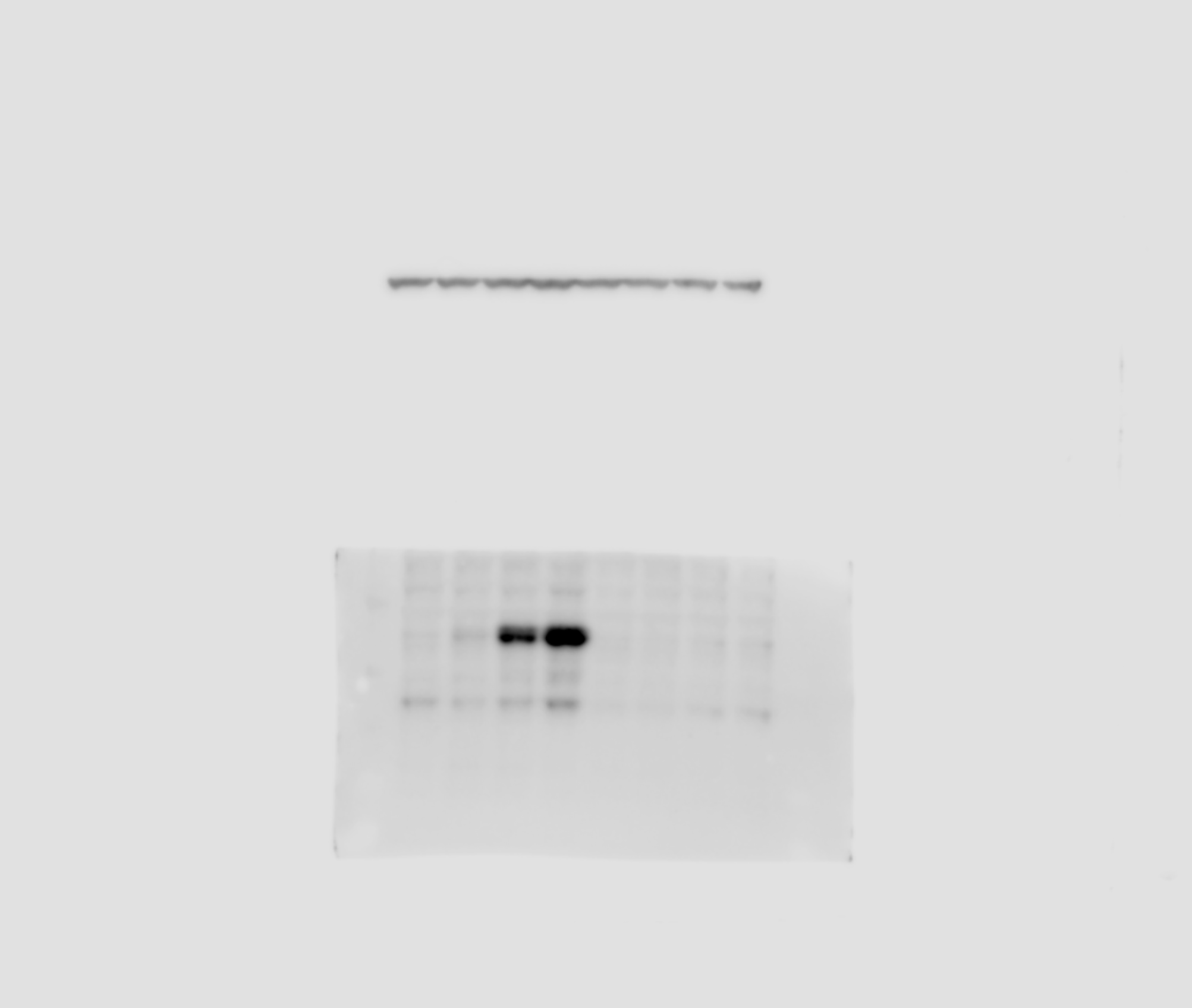

Supplement: Figure 2—source data 1. [file elife-87255-fig2-data1.zip › Figure 2D Source Data_pRab12 and GAPDH_Chemi.tif]

Figure 2A and C

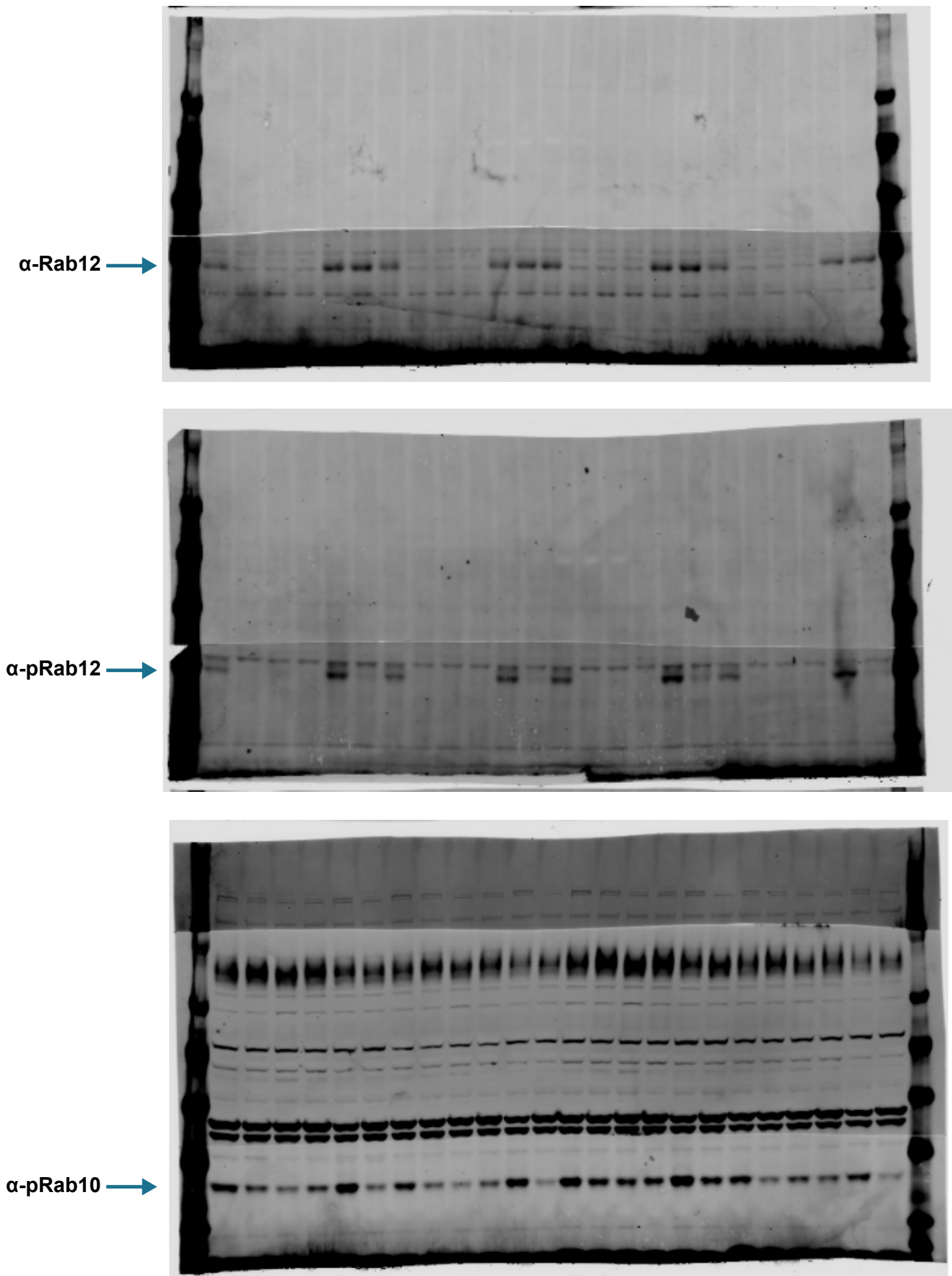

Figure 2A and C

$\alpha$ -Rab10 →

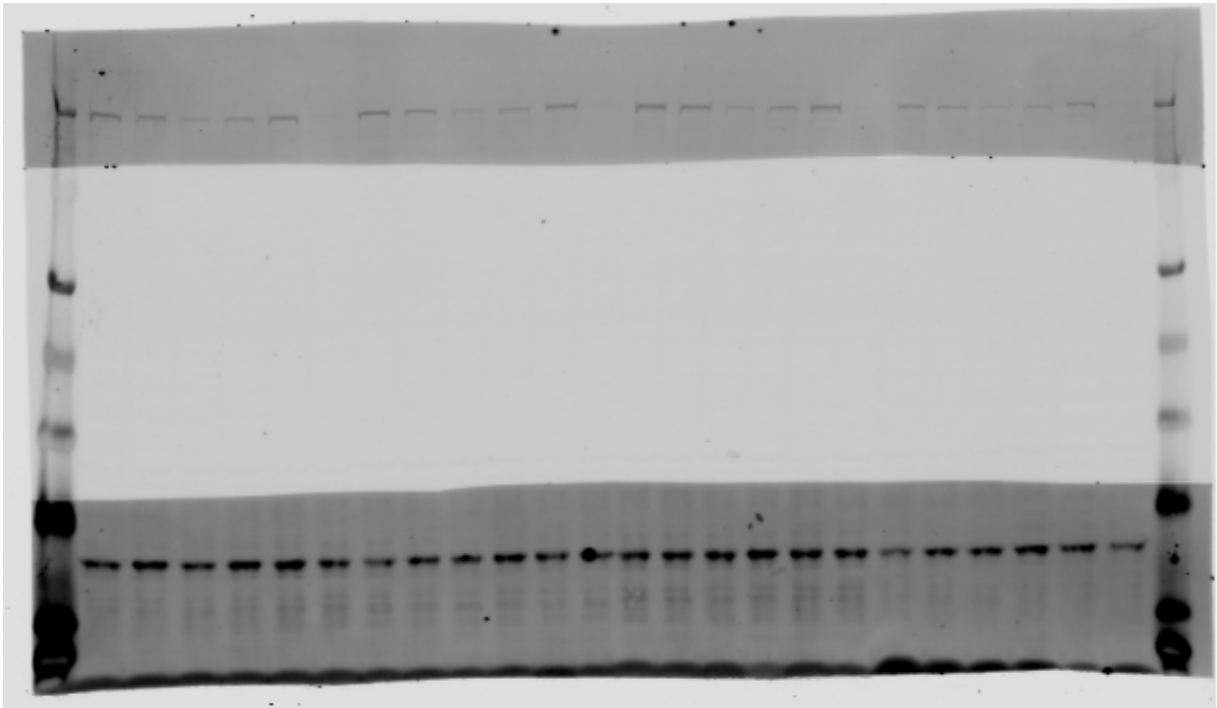

$\alpha$ -GAPDH →

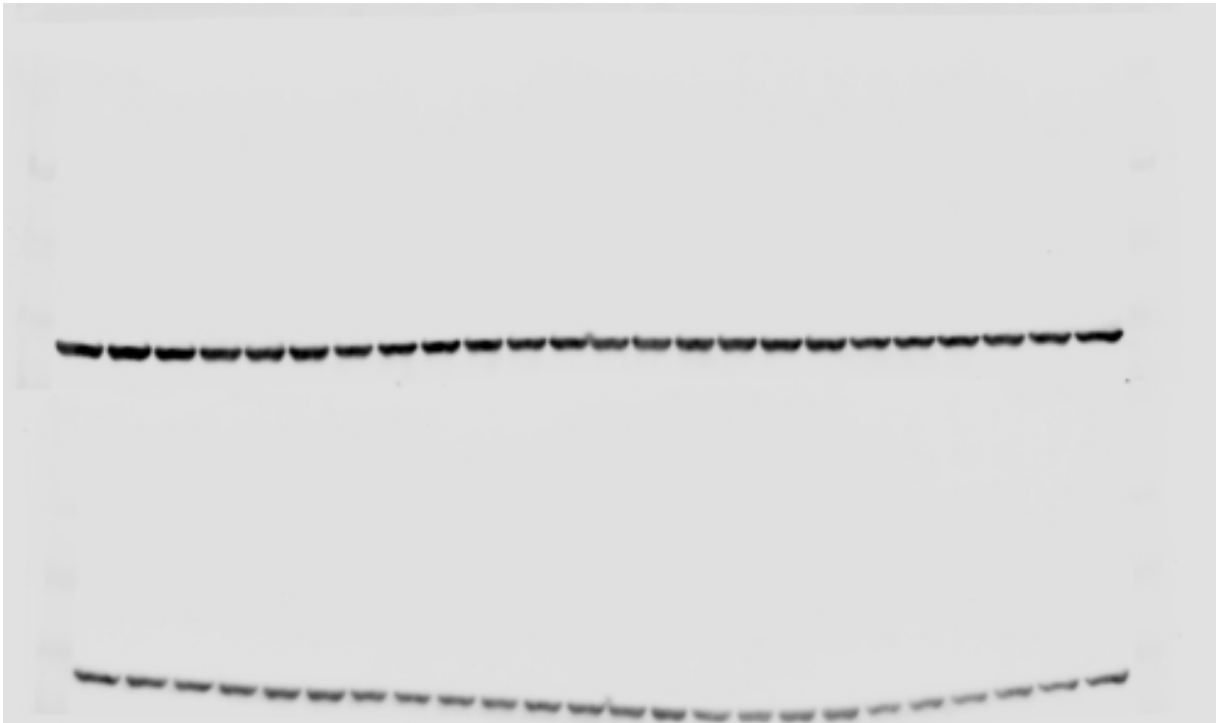

Figure 2D

$\alpha$ -Rab12 →

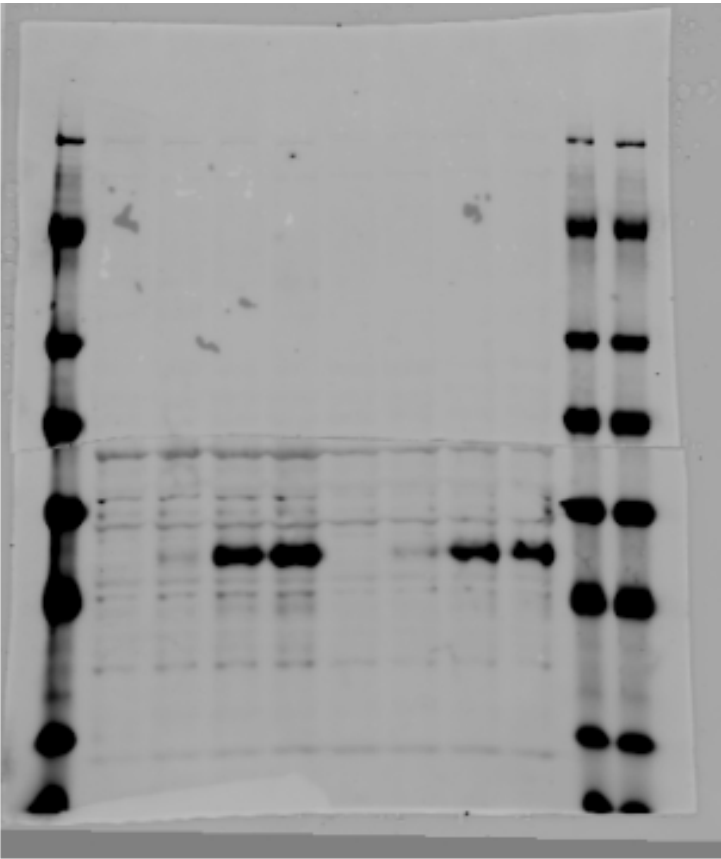

$\alpha$ -pRab12 →

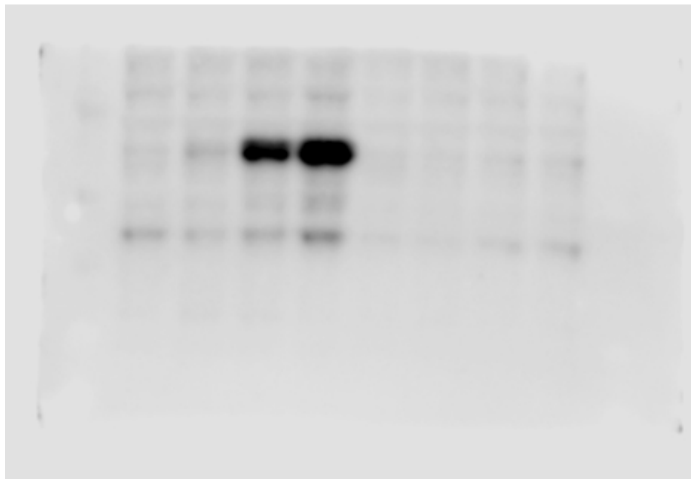

$\alpha$ -GAPDH →

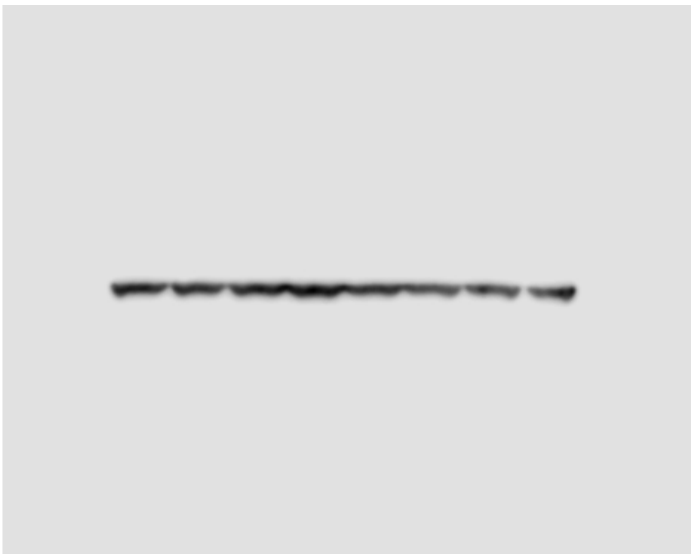

Supplement: Figure 2—source data 2. [file elife-87255-fig2-data2.pdf]

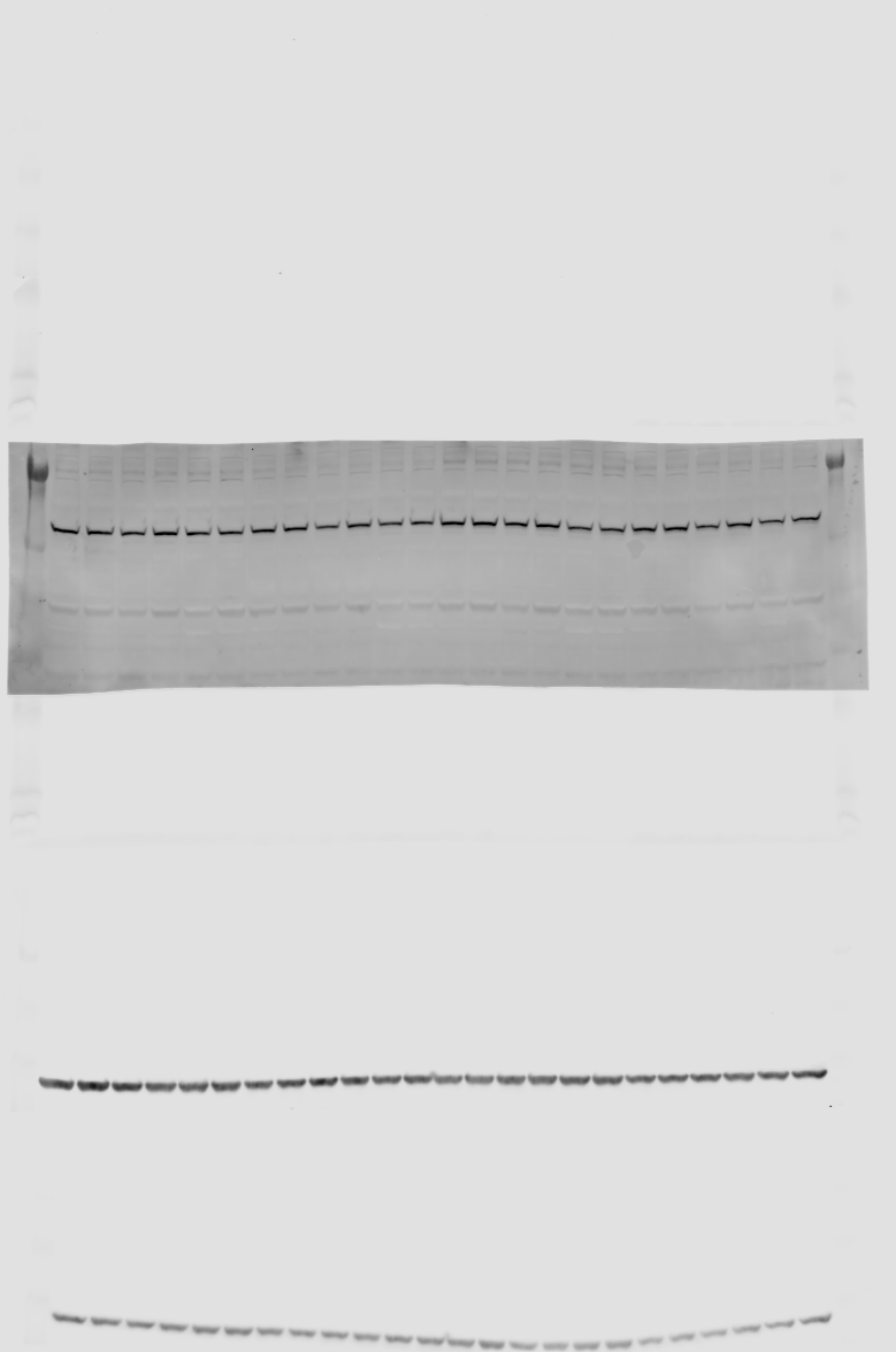

Supplement: Figure 2—figure supplement 1—source data 1. [file elife-87255-fig2-figsupp1-data1.zip › Figure 2-fig supplement 1A_GAPDH_800.tif]

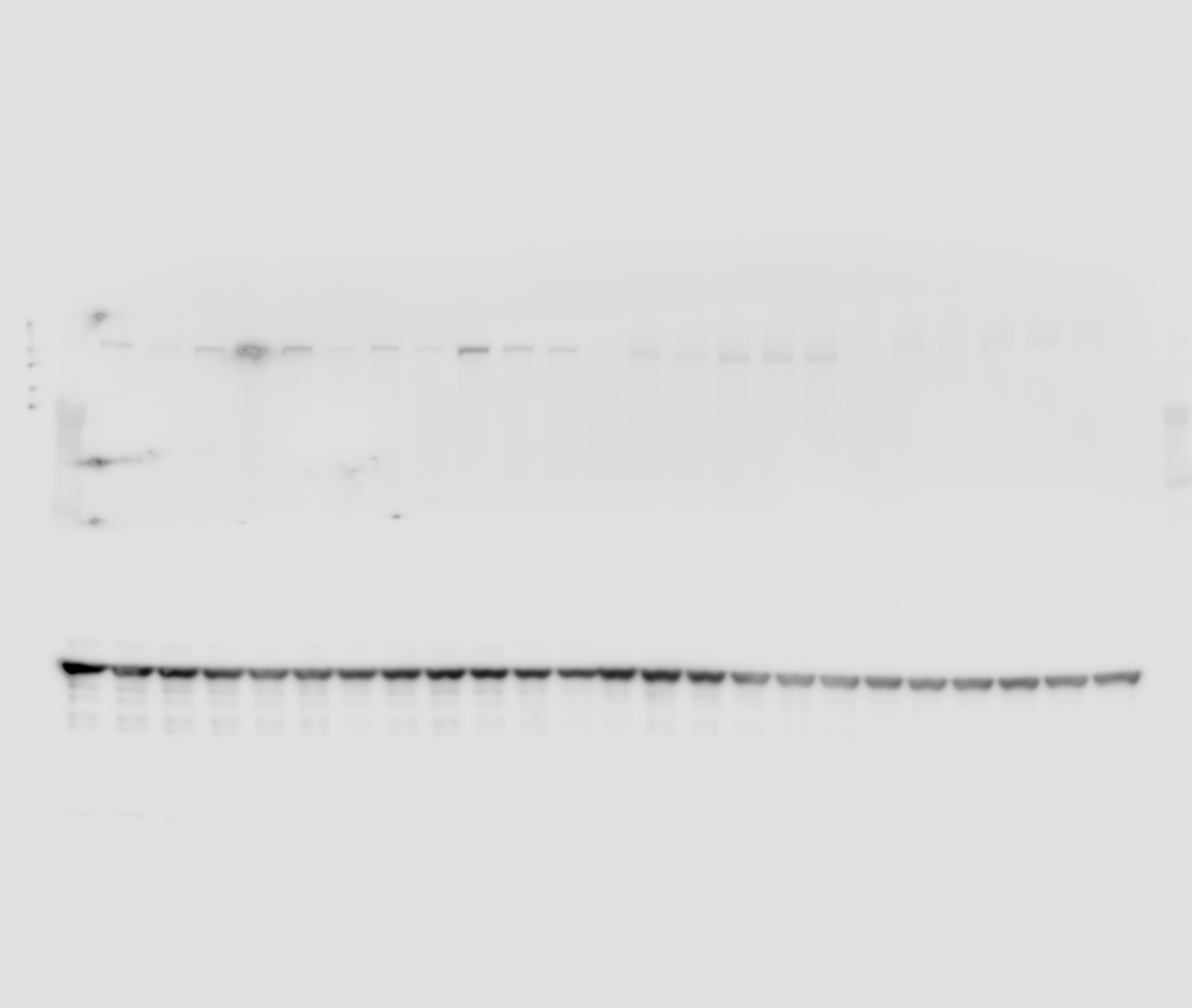

Supplement: Figure 2—figure supplement 1—source data 1. [file elife-87255-fig2-figsupp1-data1.zip › Figure 2-fig supplement 1B-E_GAPDH_Chemi.tif]

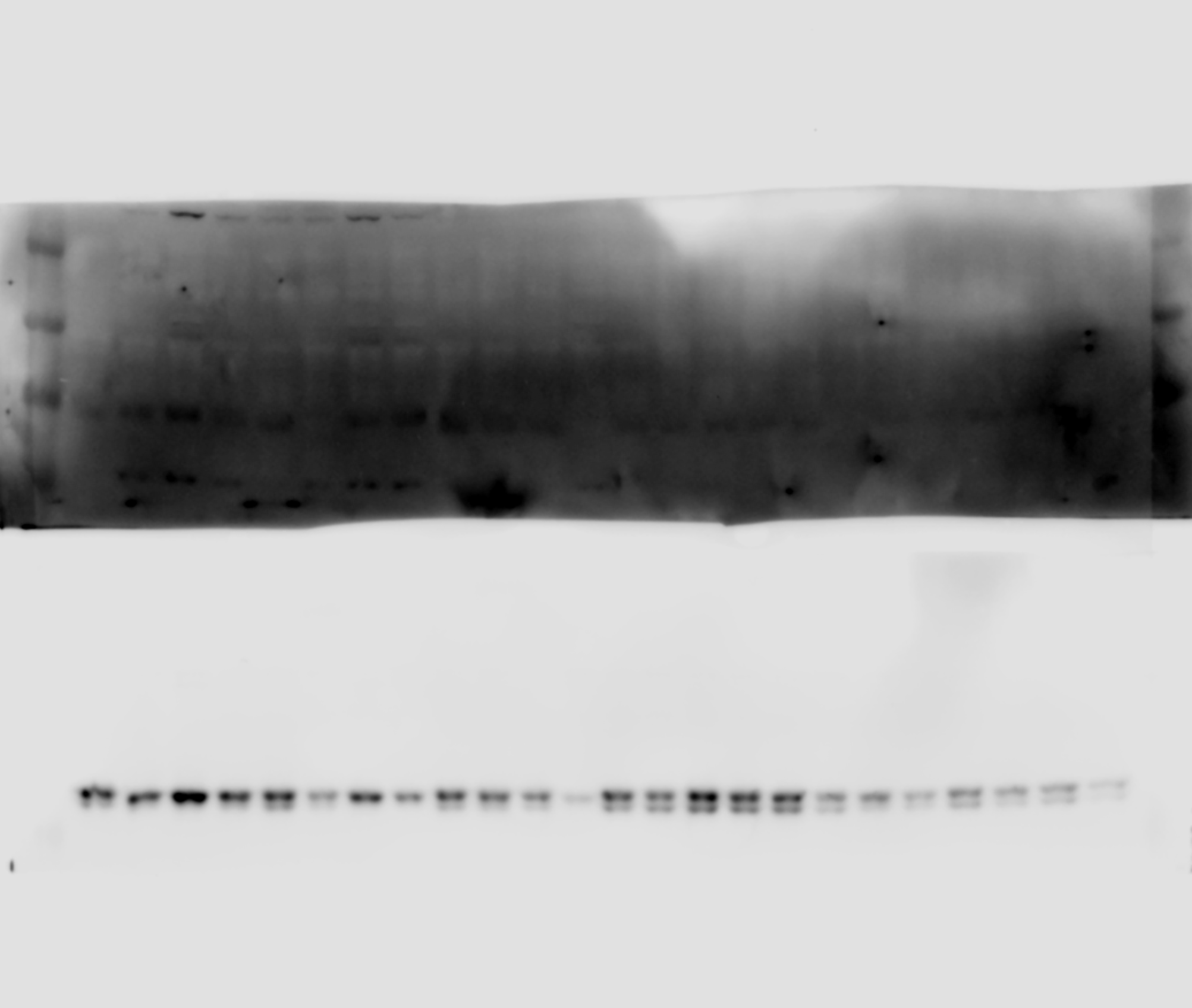

Supplement: Figure 2—figure supplement 1—source data 1. [file elife-87255-fig2-figsupp1-data1.zip › Figure 2-fig supplement 1B-E_pRab10_Chemi.tif]

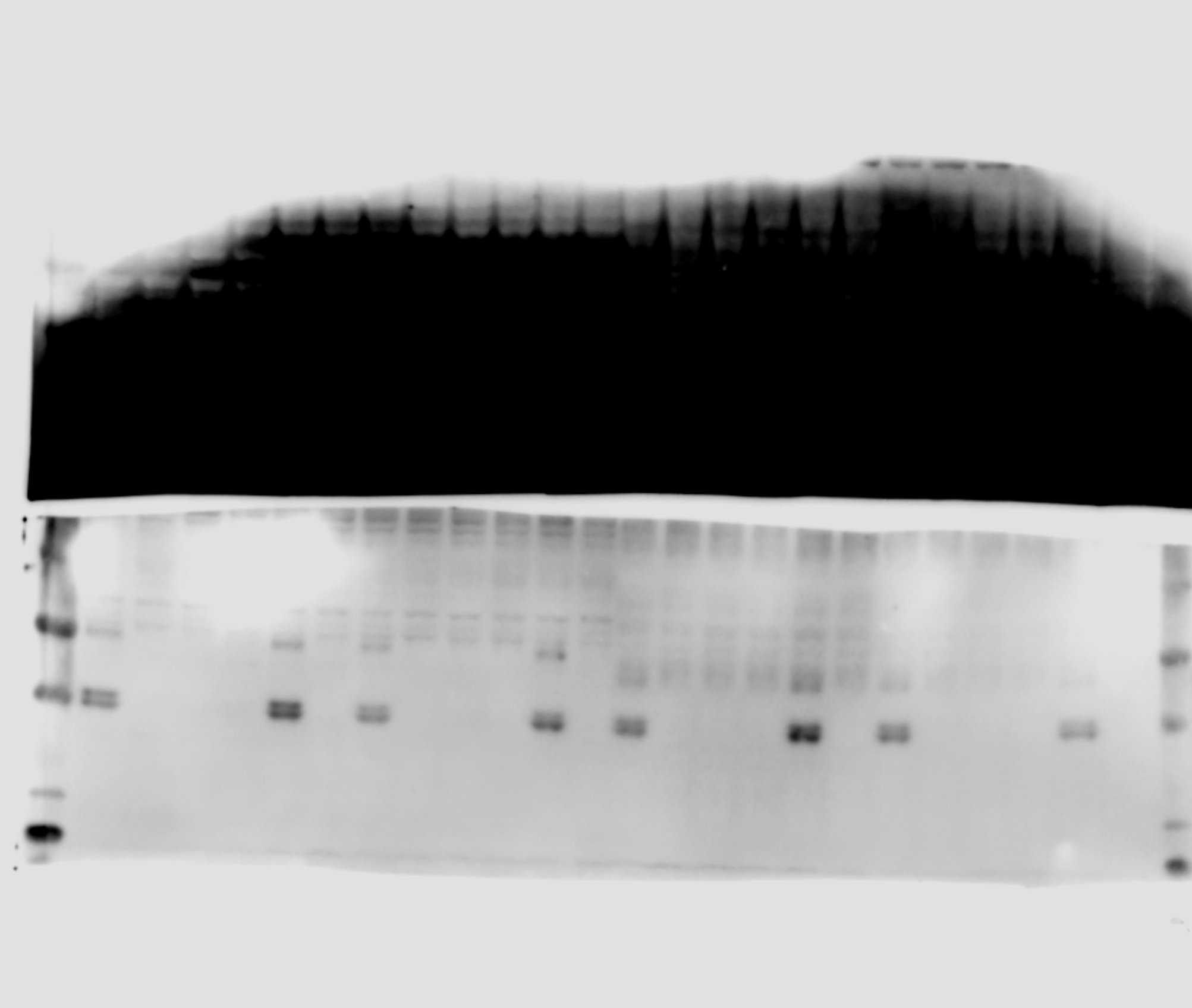

Supplement: Figure 2—figure supplement 1—source data 1. [file elife-87255-fig2-figsupp1-data1.zip › Figure 2-fig supplement 1B-E_pRab12_Chemi.tif]

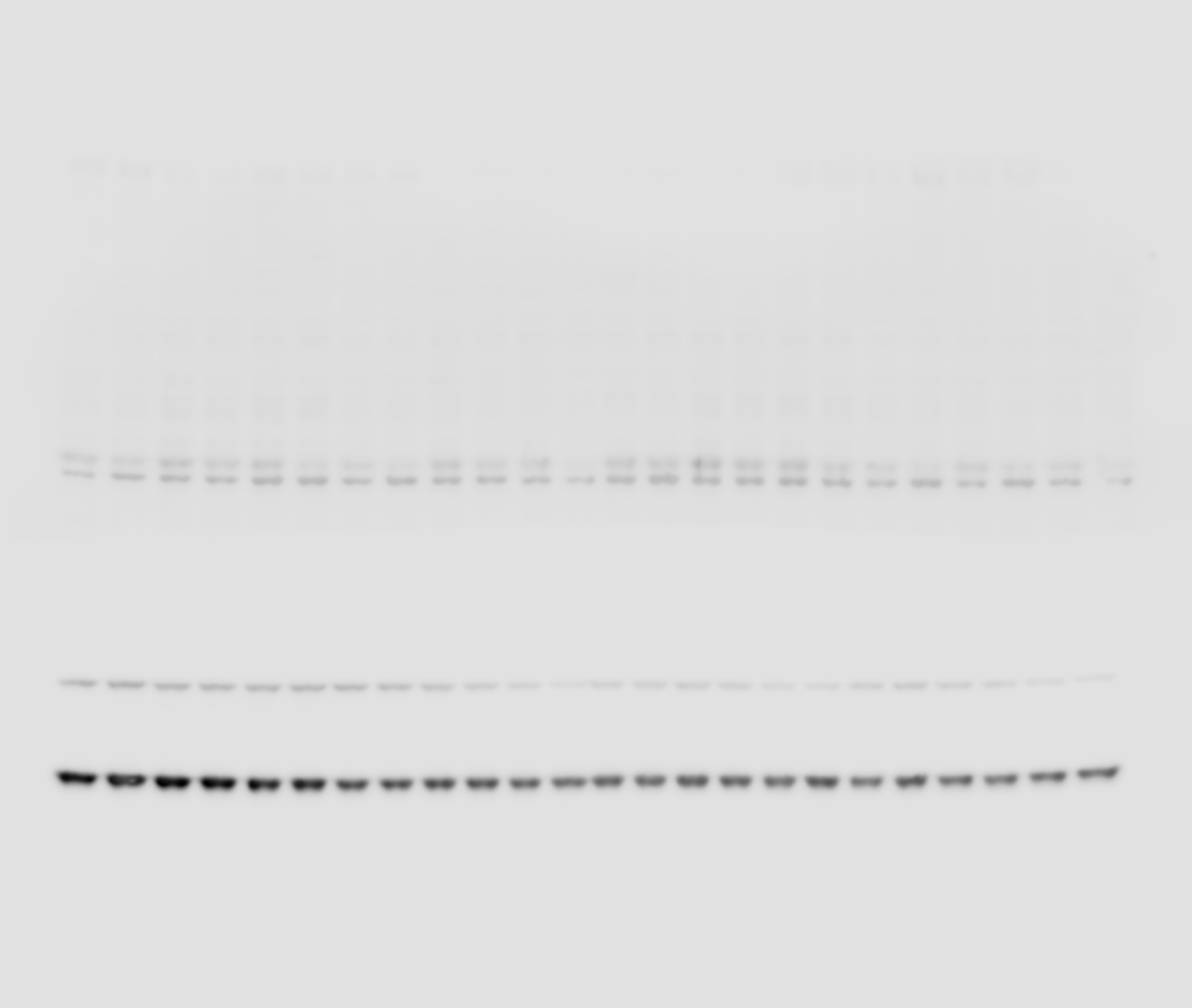

Supplement: Figure 2—figure supplement 1—source data 1. [file elife-87255-fig2-figsupp1-data1.zip › Figure 2-fig supplement 1B-E_Rab10_Chemi.tif]

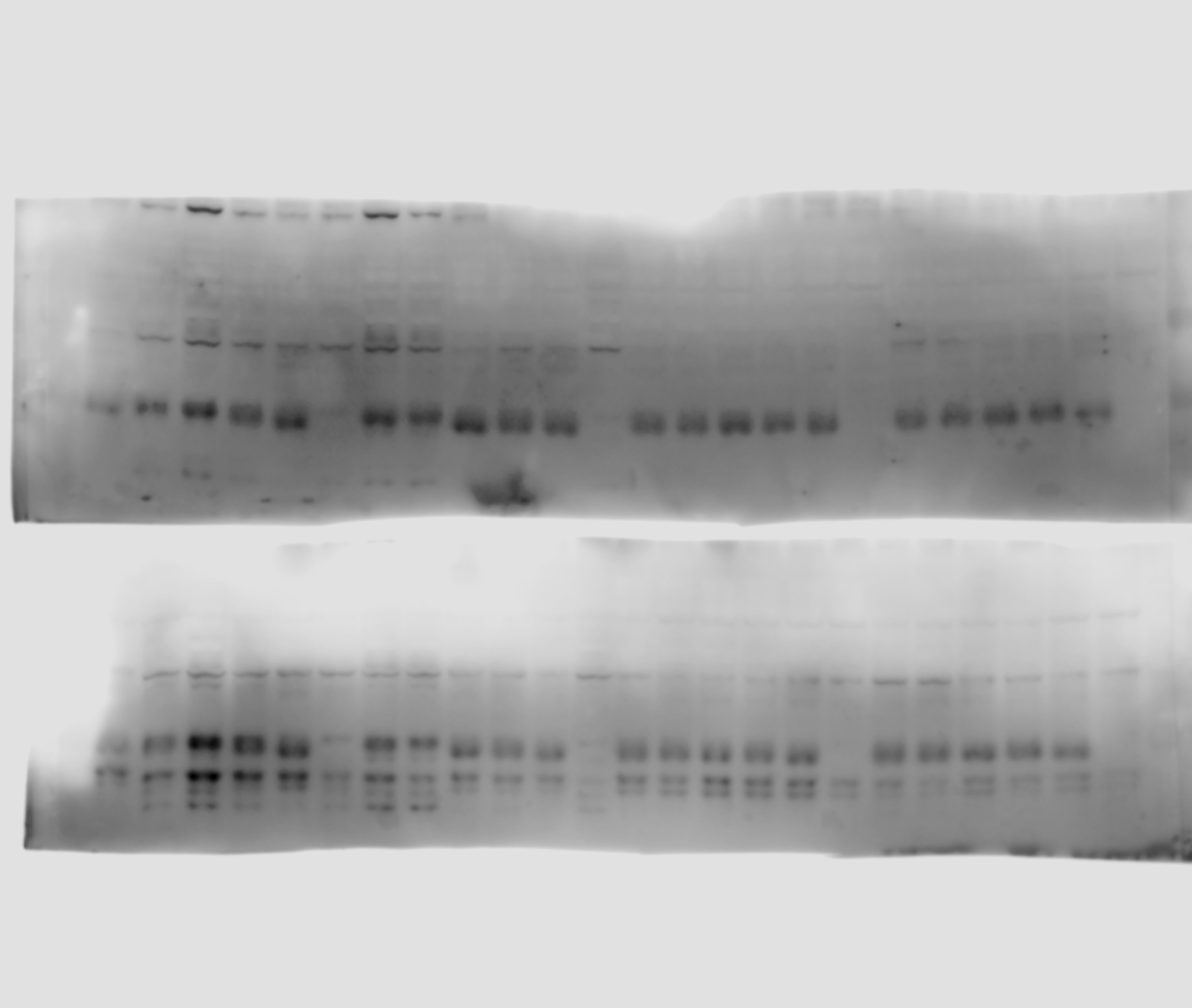

Supplement: Figure 2—figure supplement 1—source data 1. [file elife-87255-fig2-figsupp1-data1.zip › Figure 2-fig supplement 1B-E_Rab12_Chemi.tif]

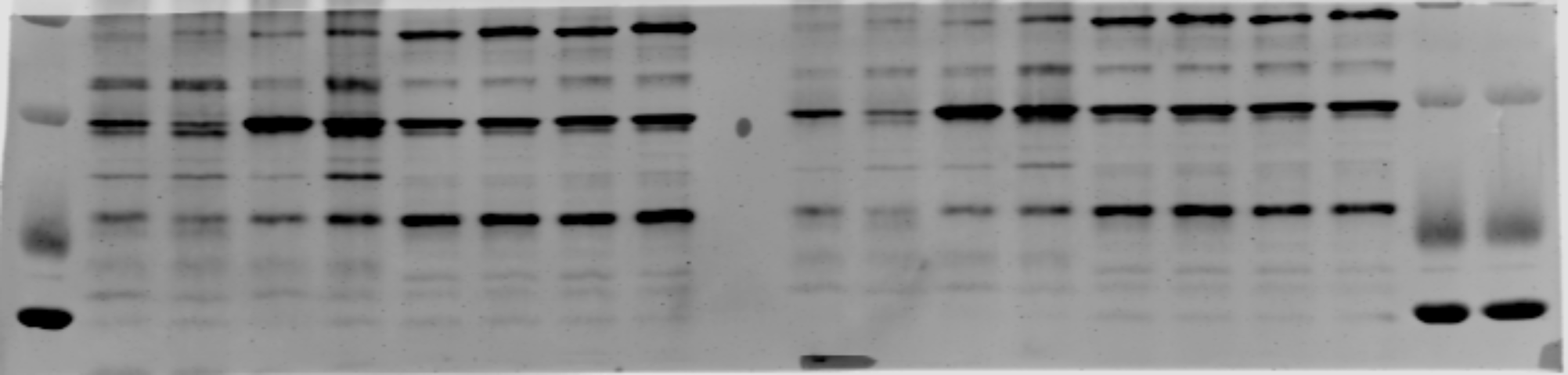

Supplement: Figure 3—source data 1. [file elife-87255-fig3-data1.zip › Figure 3D Source Data_Rab12_800_5 and 6.tif]

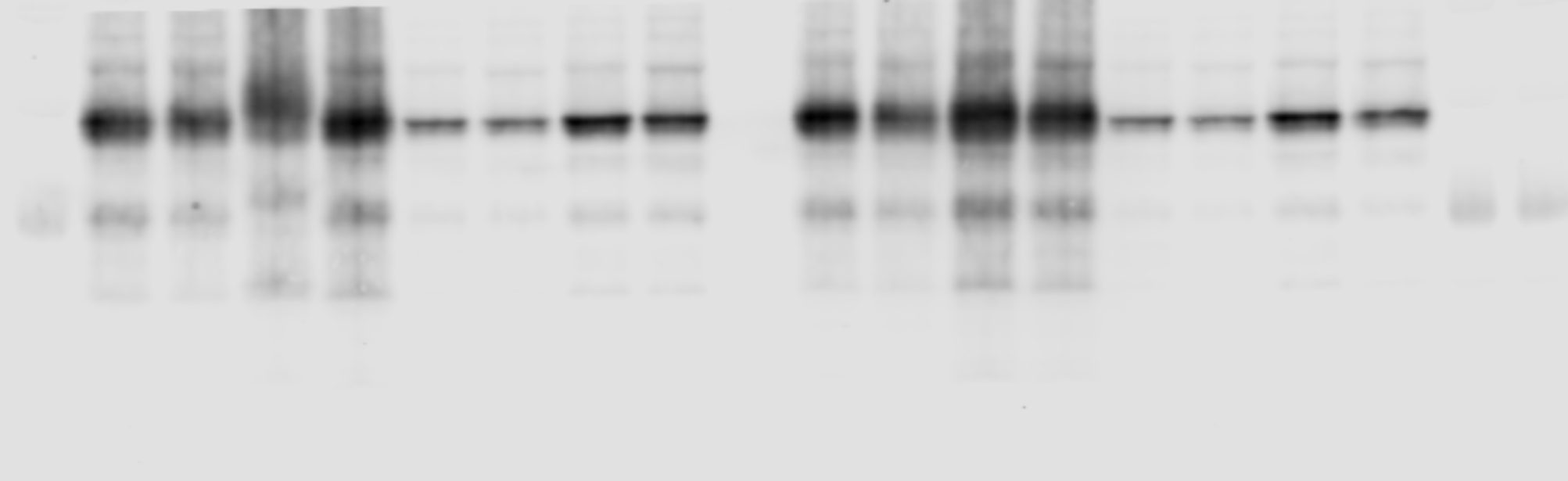

Supplement: Figure 3—source data 1. [file elife-87255-fig3-data1.zip › Figure 3A and B Source Data_HA_800_1 and 2.tif]

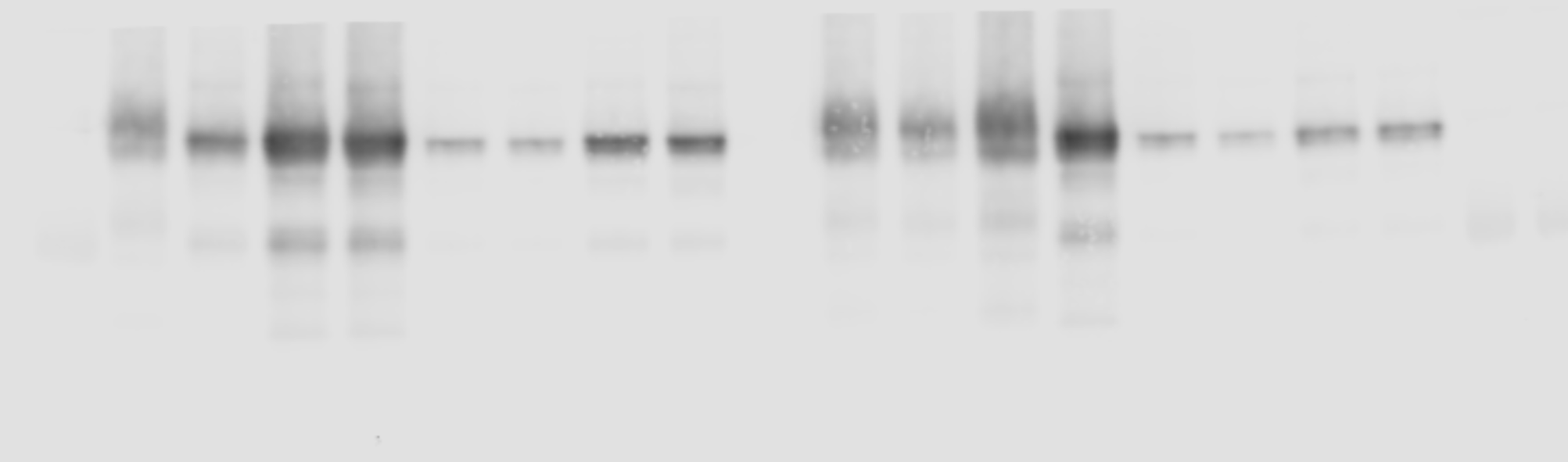

Supplement: Figure 3—source data 1. [file elife-87255-fig3-data1.zip › Figure 3A and B Source Data_HA_800_3 and 4.tif]

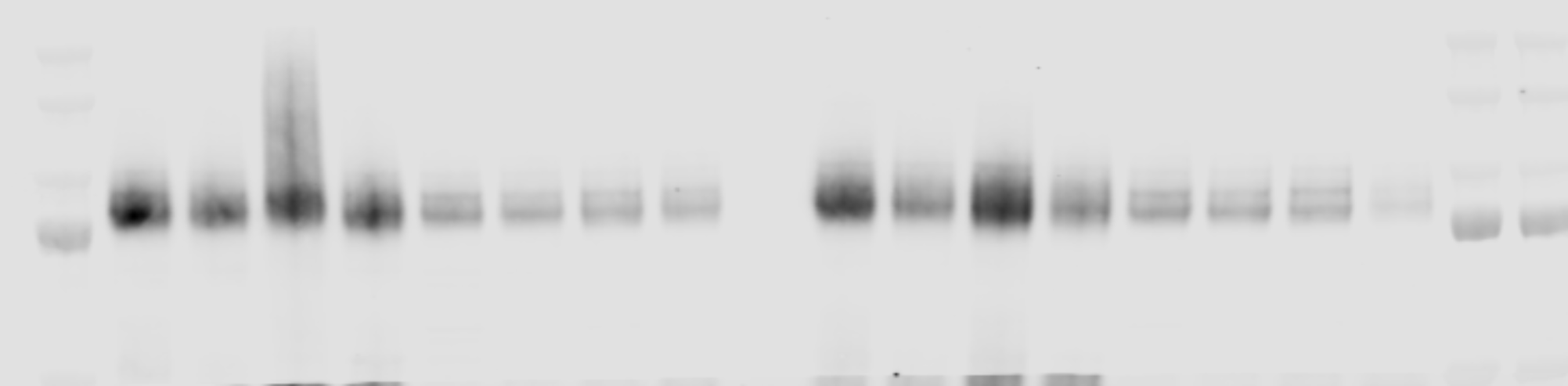

Supplement: Figure 3—source data 1. [file elife-87255-fig3-data1.zip › Figure 3A and B Source Data_LAMP1_700_1 and 2.tif]

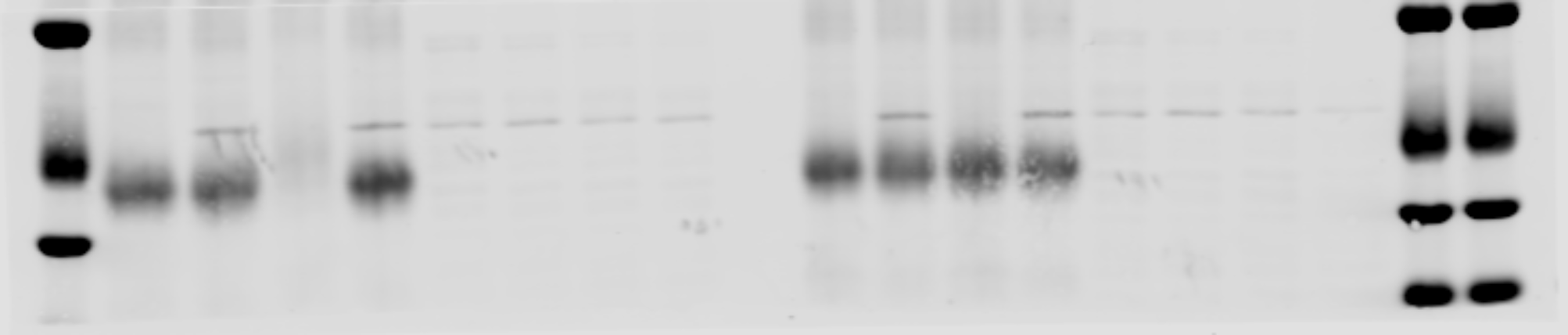

Supplement: Figure 3—source data 1. [file elife-87255-fig3-data1.zip › Figure 3A and B Source Data_pRab12 and Gal3_700_1 and 2.tif]

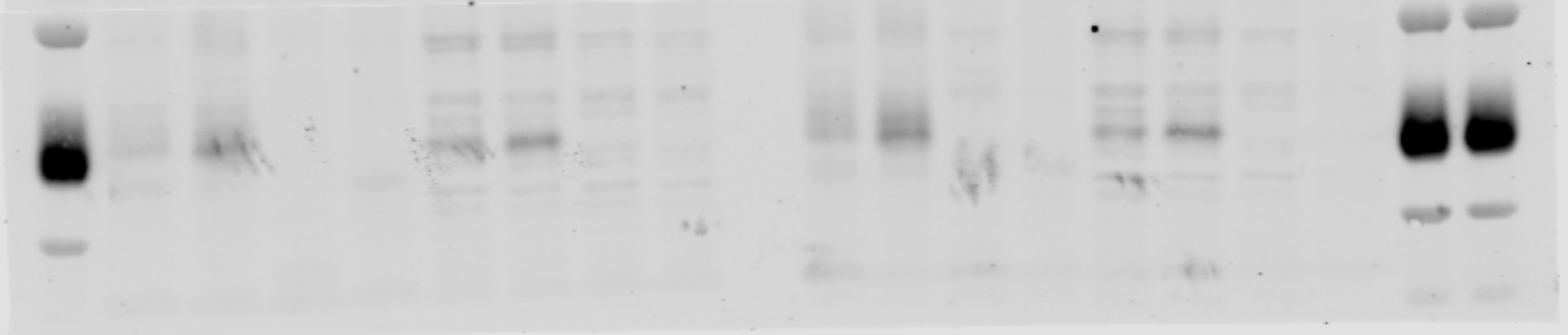

Supplement: Figure 3—source data 1. [file elife-87255-fig3-data1.zip › Figure 3A and B Source Data_pRab12 and Gal3_800_1 and 2.tif]

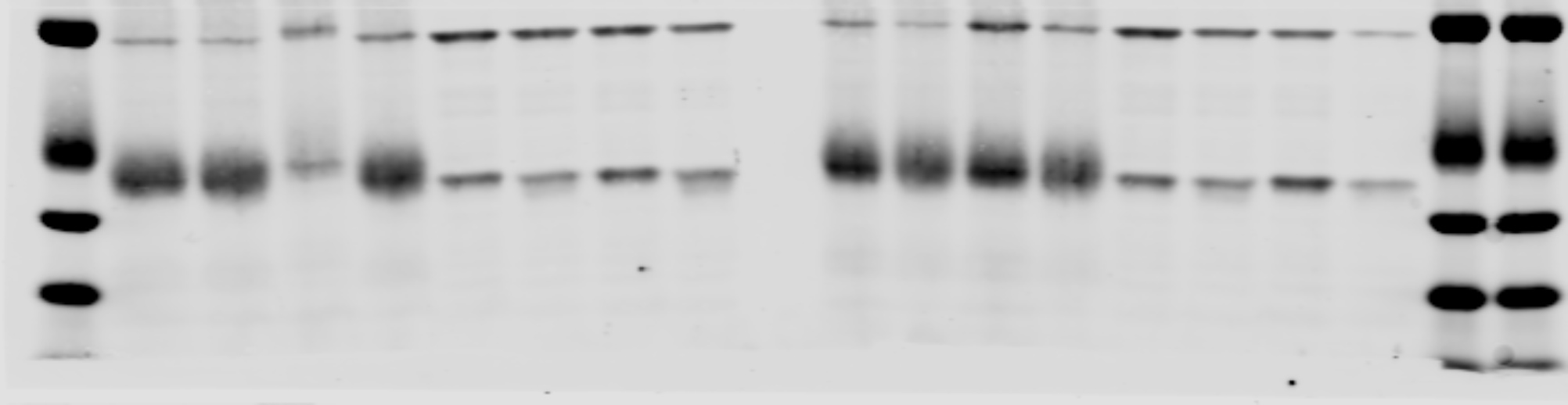

Supplement: Figure 3—source data 1. [file elife-87255-fig3-data1.zip › Figure 3A and B Source Data_total Rab10 and pRab10_700_1 and 2.tif]

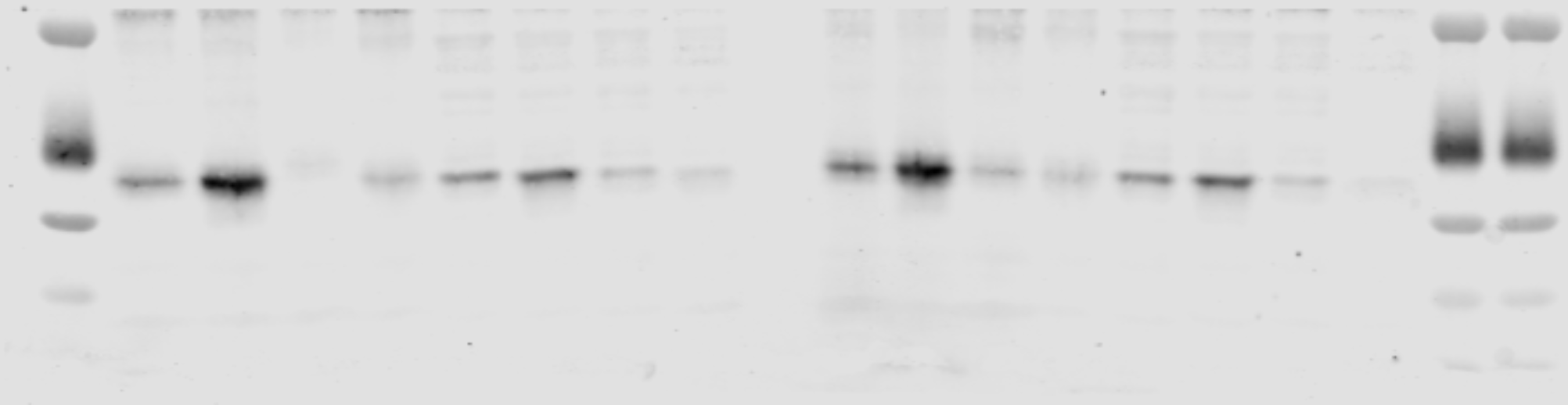

Supplement: Figure 3—source data 1. [file elife-87255-fig3-data1.zip › Figure 3A and B Source Data_total Rab10 and pRab10_800_1 and 2.tif]

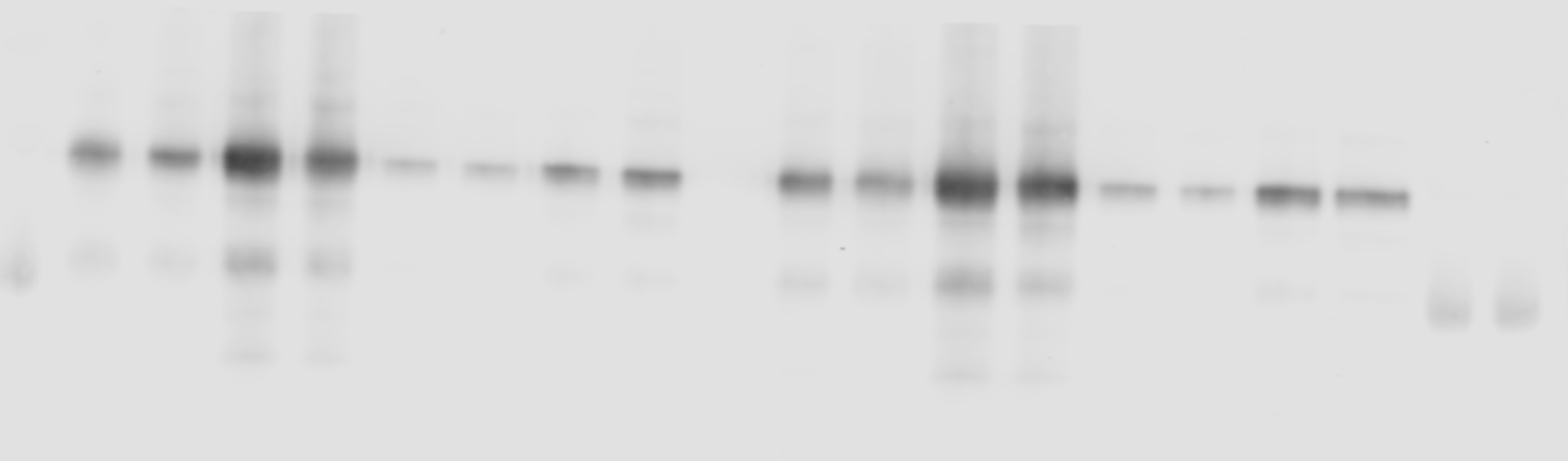

Supplement: Figure 3—source data 1. [file elife-87255-fig3-data1.zip › Figure 3D Source Data_HA_800_5 and 6.tif]

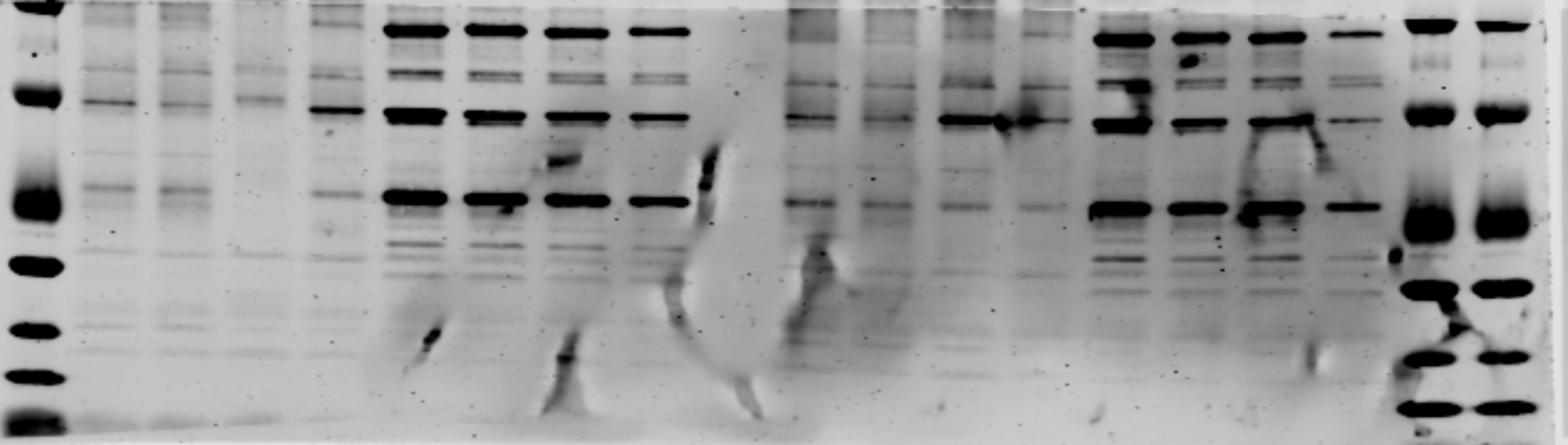

Supplement: Figure 3—source data 1. [file elife-87255-fig3-data1.zip › Figure 3D Source Data_Rab12_800_1 and 2.tif]

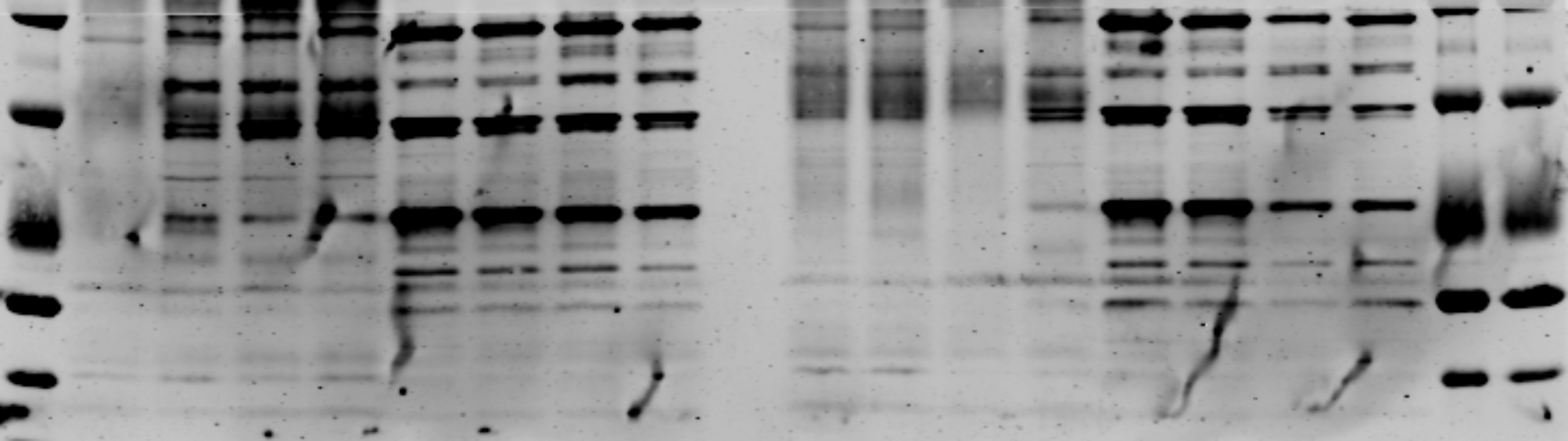

Supplement: Figure 3—source data 1. [file elife-87255-fig3-data1.zip › Figure 3D Source Data_Rab12_800_3 and 4.tif]

Figure 3A+3B

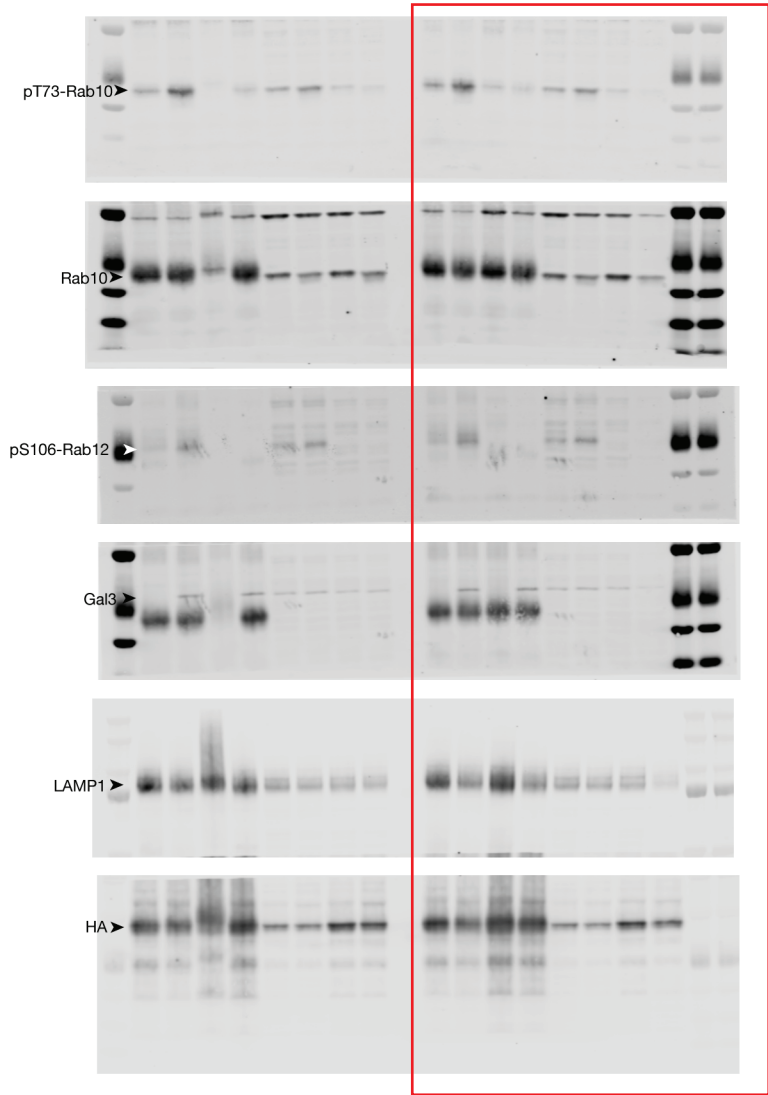

3A example blot replicate

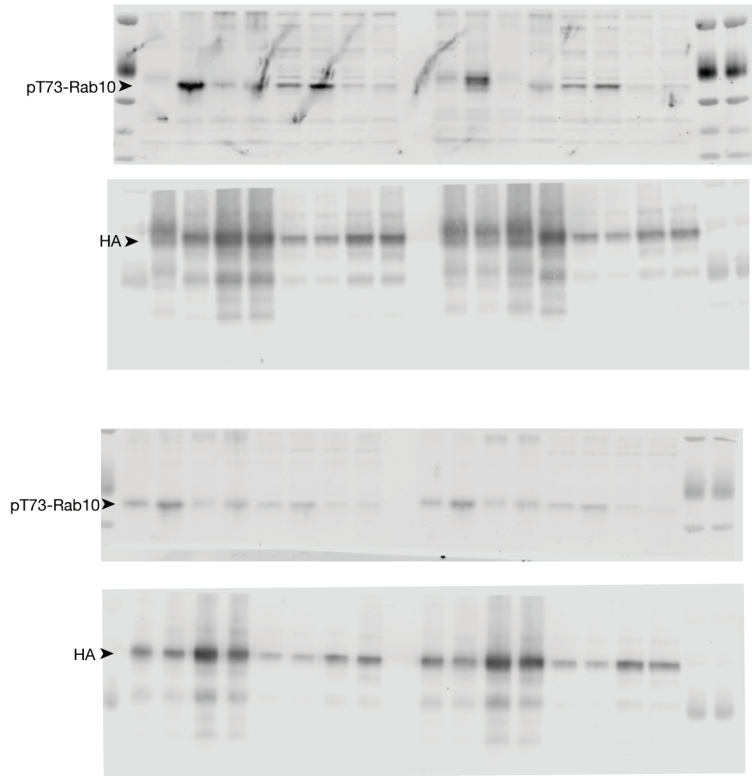

**Figure 3D**

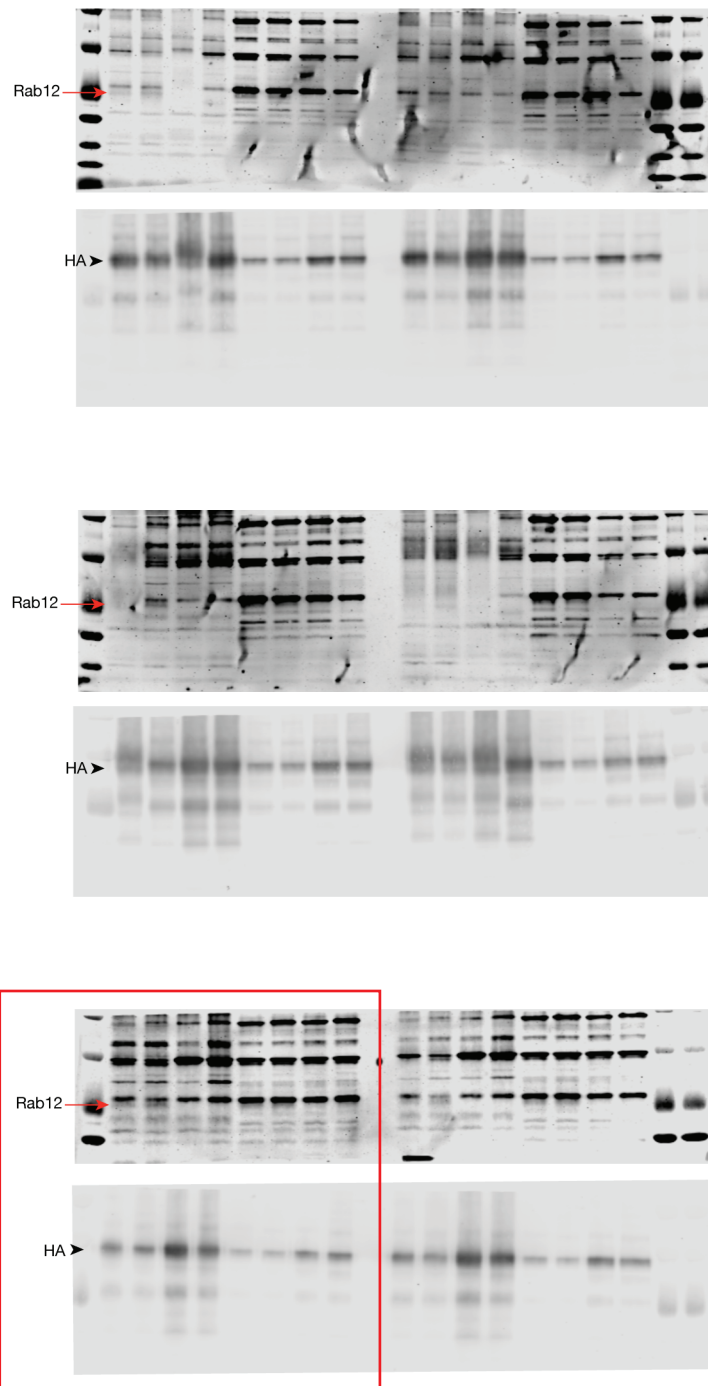

3D example blot replicate

Supplement: Figure 3—source data 2. [file elife-87255-fig3-data2.pdf]

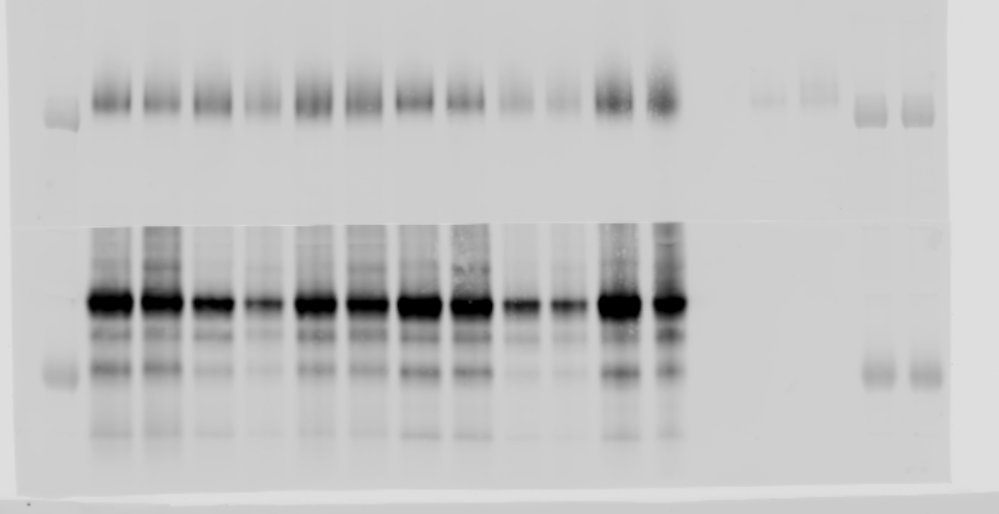

Supplement: Figure 4—source data 1. [file elife-87255-fig4-data1.zip › Raw Blots for Figure 4/Figure 4D_LAMP1_HA_800_6-7.png]
