# Supplementary material for: Rab12 is a regulator of LRRK2 and its activation by damaged lysosomes
Source: eLife. 2023 Oct 24;12:e87255. doi: 10.7554/eLife.87255 (PMC10708889; doi:10.7554/eLife.87255)

Figure 1D

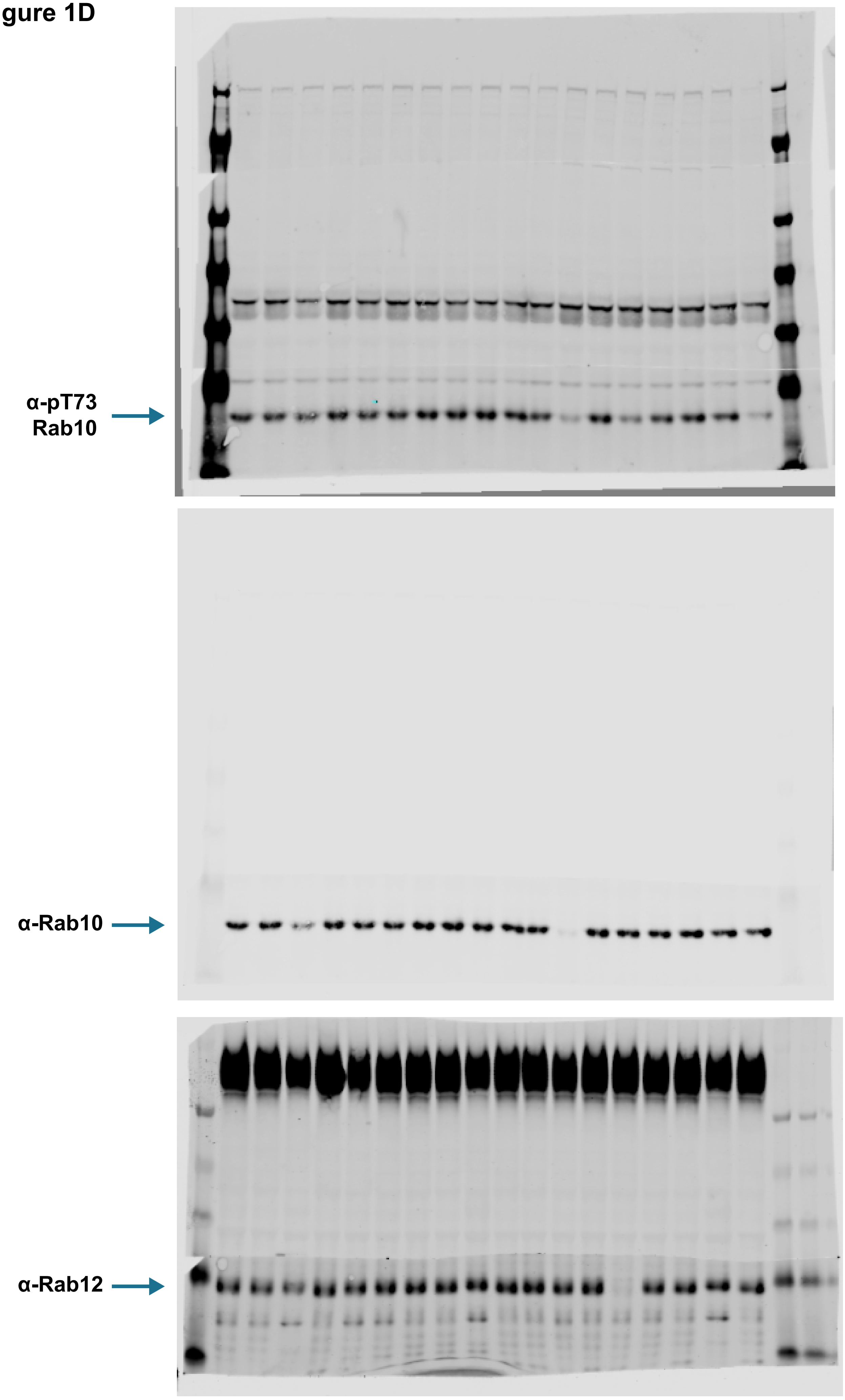

Figure 1D

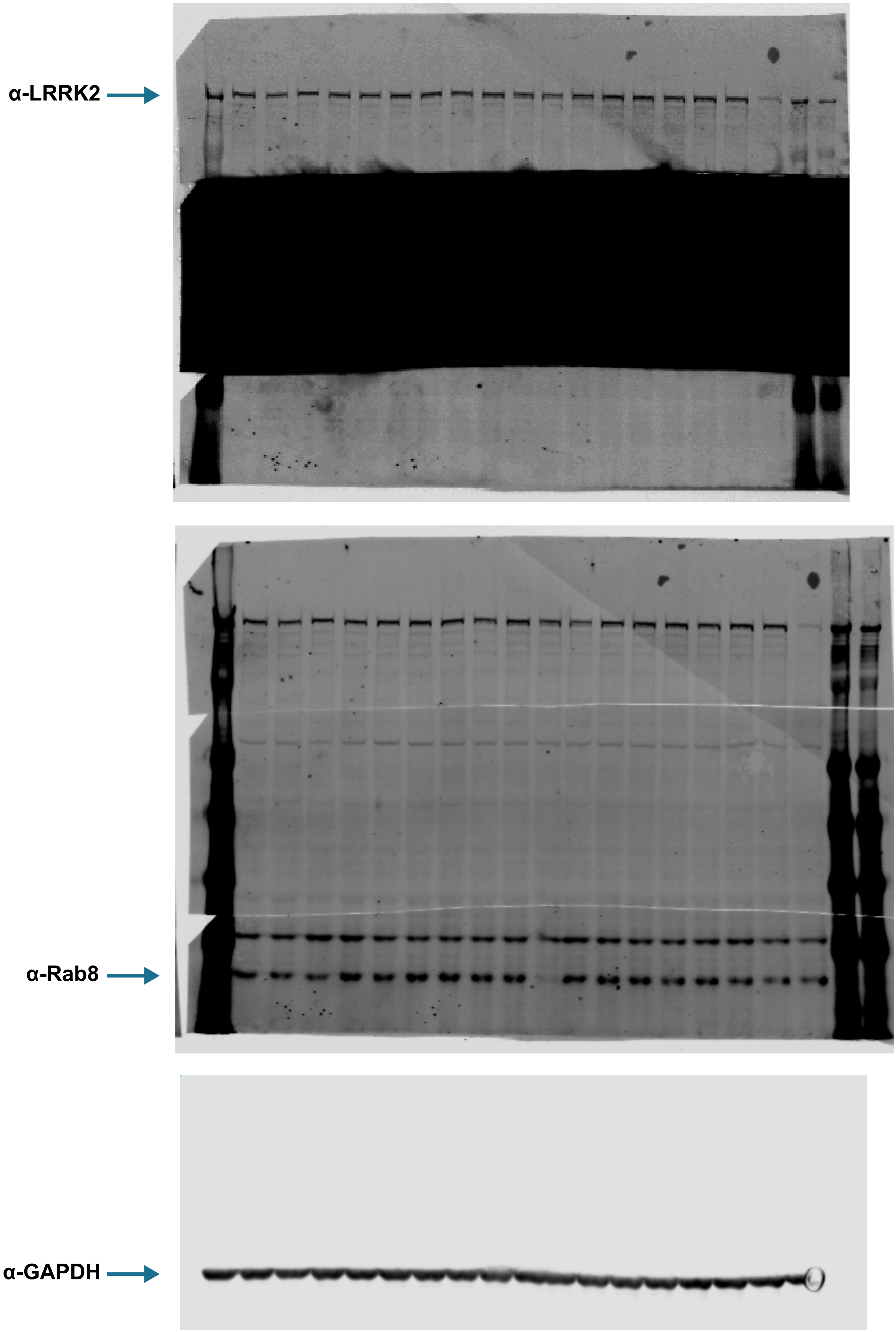

Figure 1E

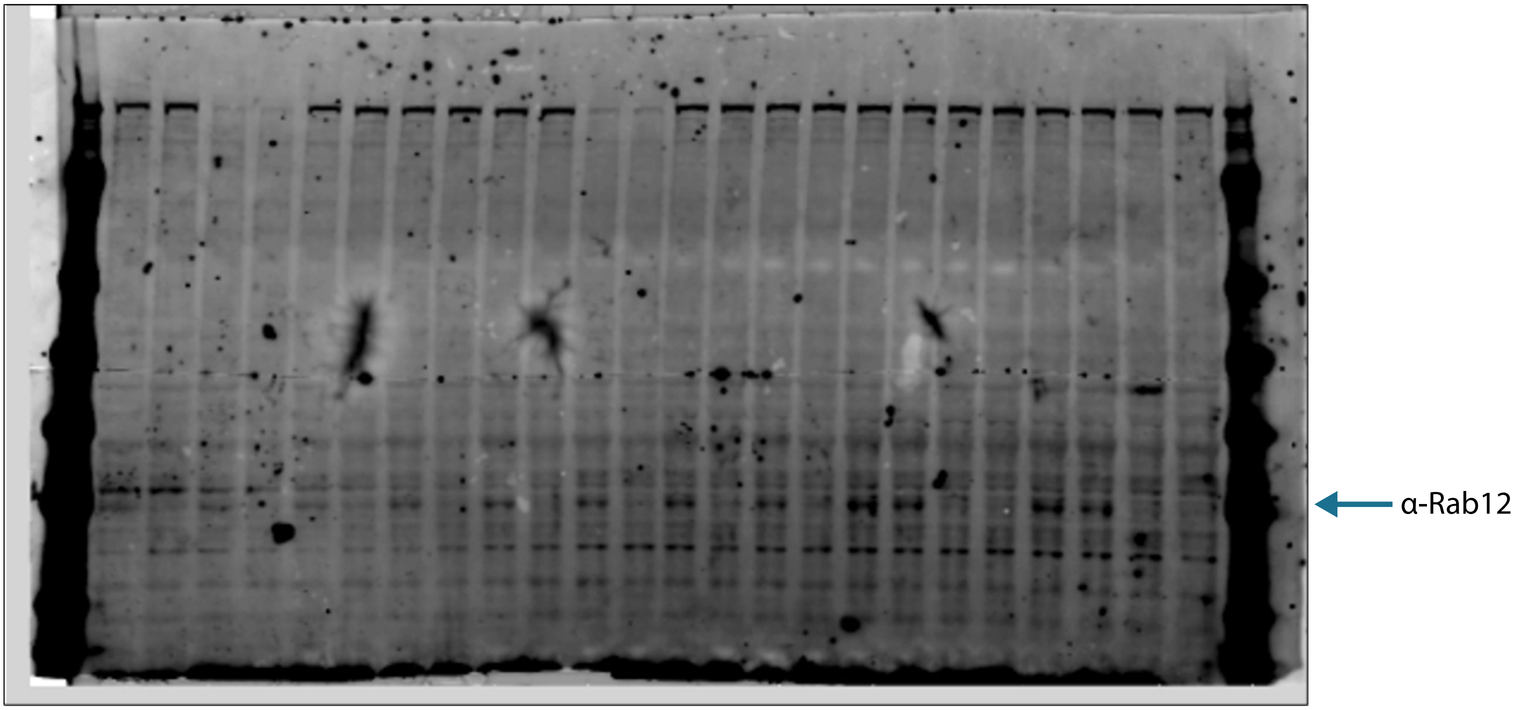

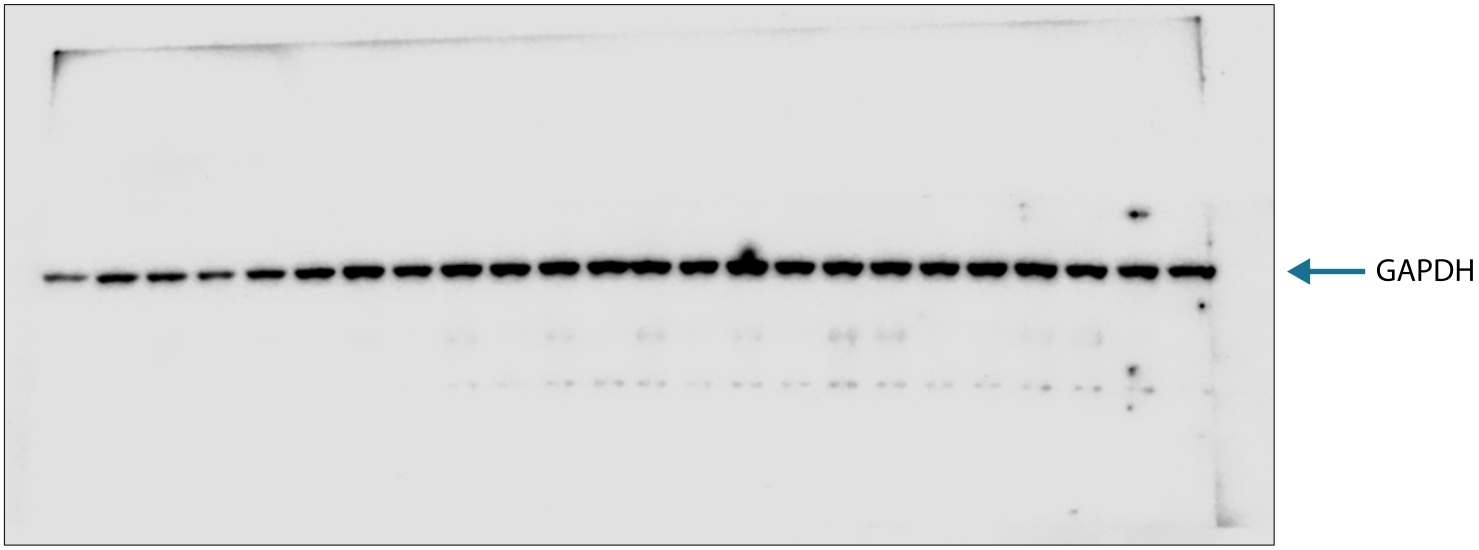

Figure 1F

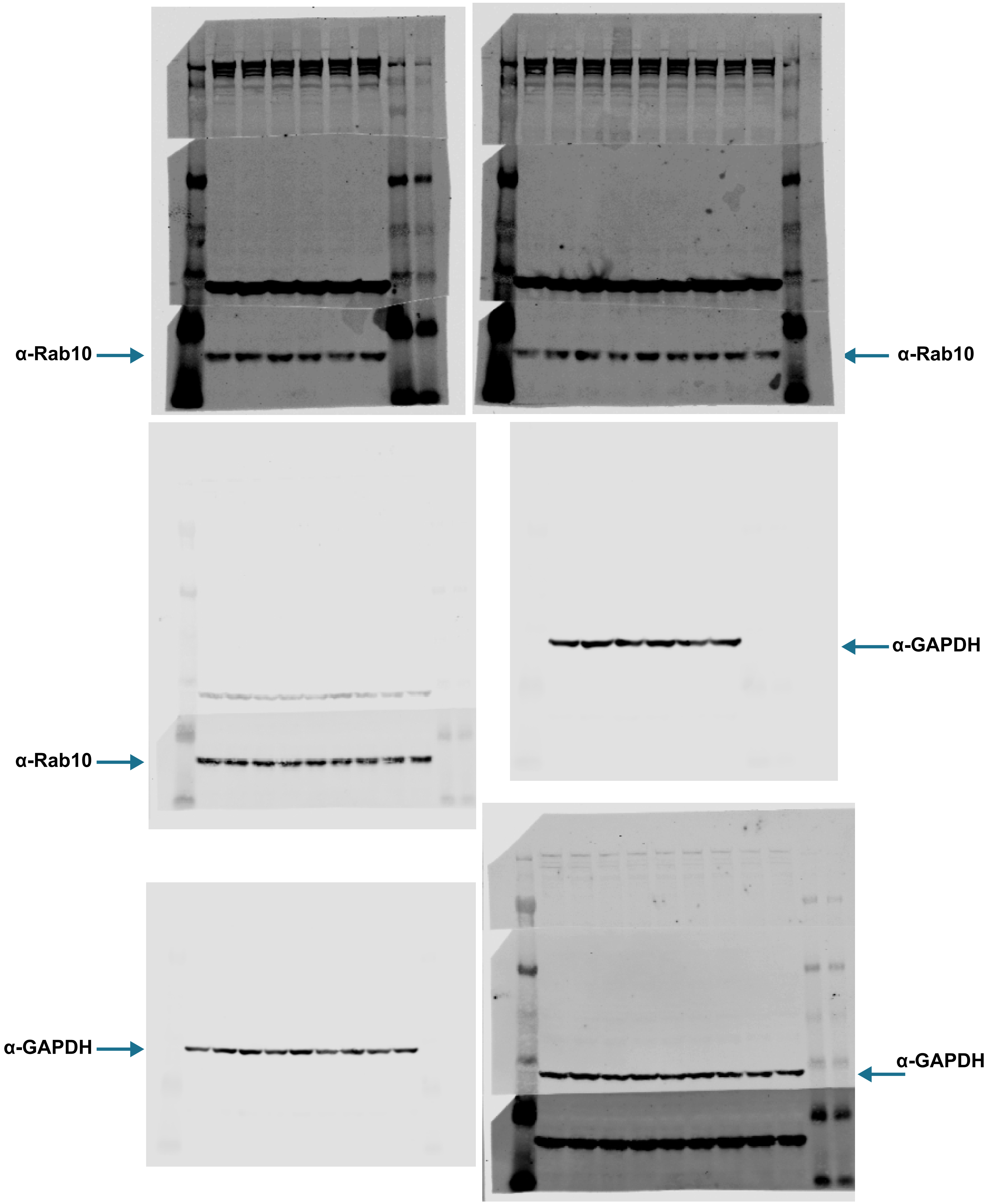

Supplement: Figure 1—source data 2. [file elife-87255-fig1-data2.pdf]
