# Supplementary material for: Rab12 is a regulator of LRRK2 and its activation by damaged lysosomes
Source: eLife. 2023 Oct 24;12:e87255. doi: 10.7554/eLife.87255 (PMC10708889; doi:10.7554/eLife.87255)

SI Figure 2A

Blots also correspond to representative blot shown in Figure 2A

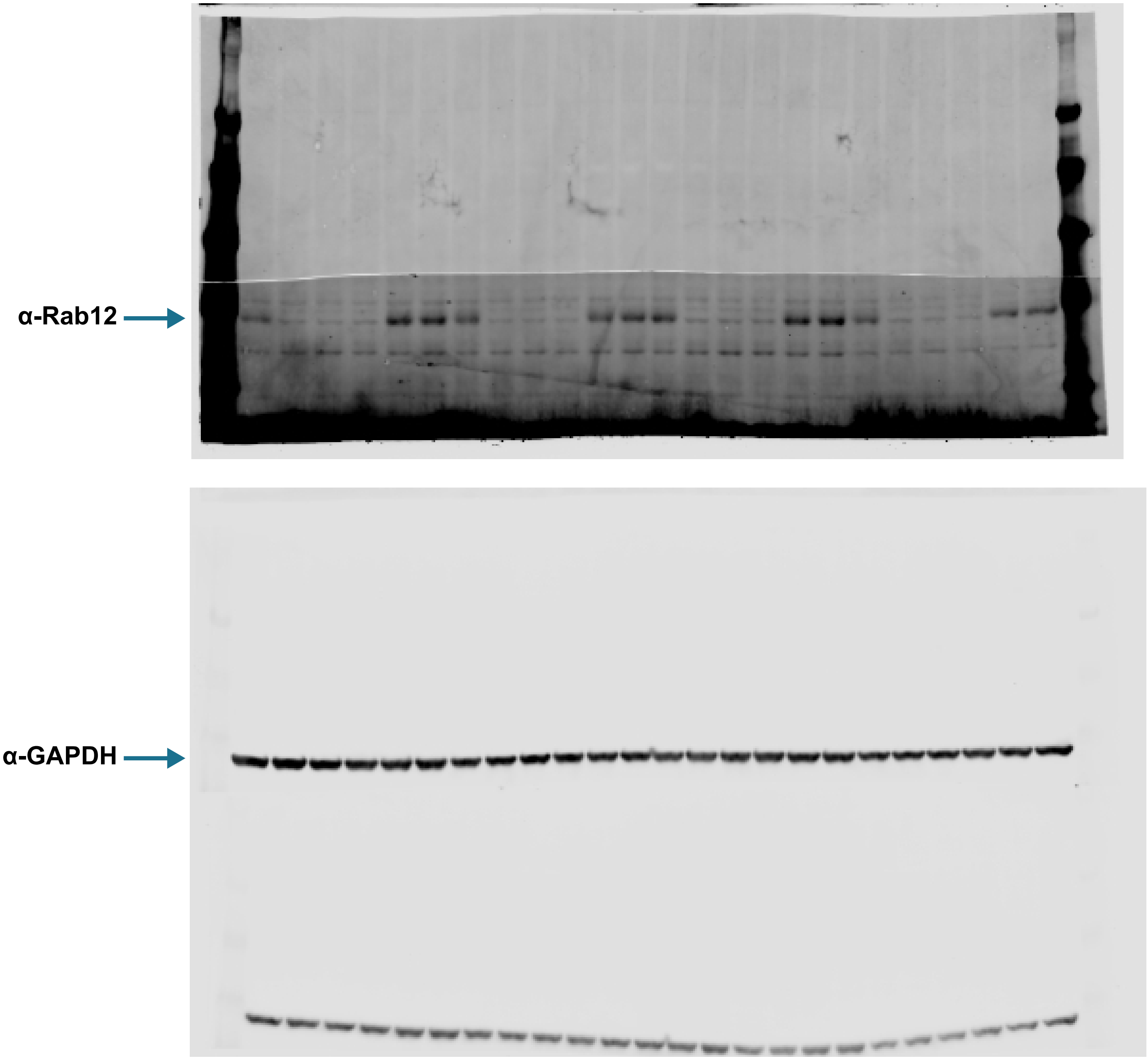

SI Figure 2B-E

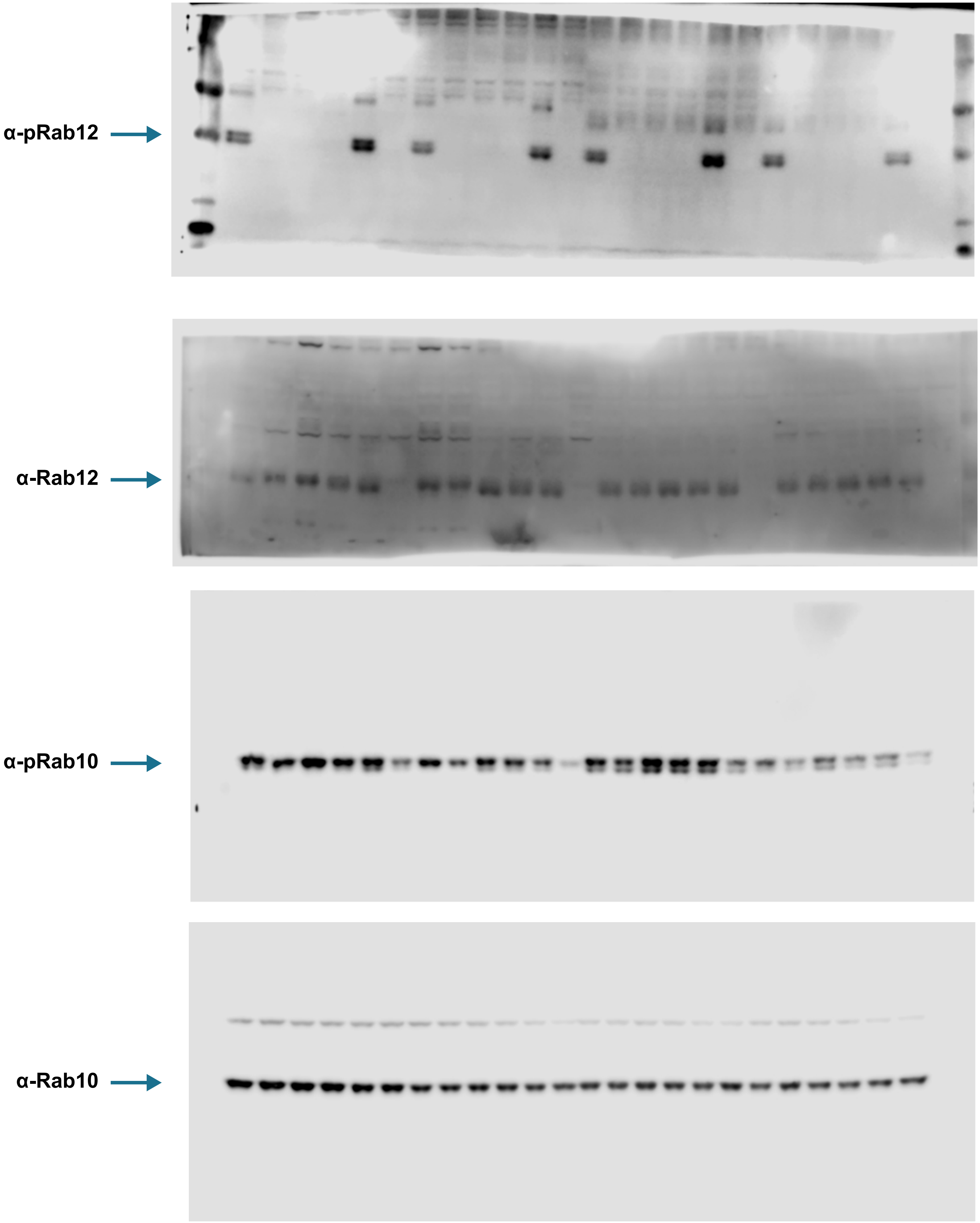

SI Figure 2B-E

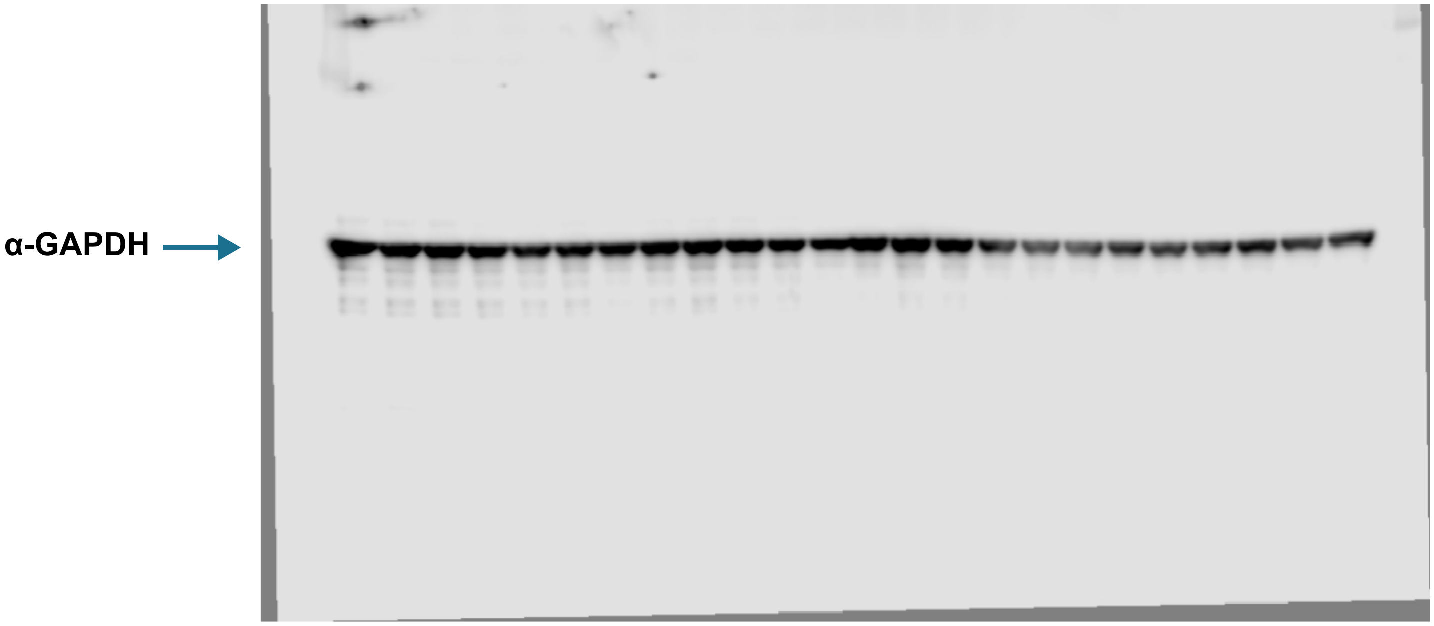

Supplement: Figure 2—figure supplement 1—source data 2. [file elife-87255-fig2-figsupp1-data2.pdf]
