# Supplementary material for: Rab12 is a regulator of LRRK2 and its activation by damaged lysosomes
Source: eLife. 2023 Oct 24;12:e87255. doi: 10.7554/eLife.87255 (PMC10708889; doi:10.7554/eLife.87255)

Figure 4A

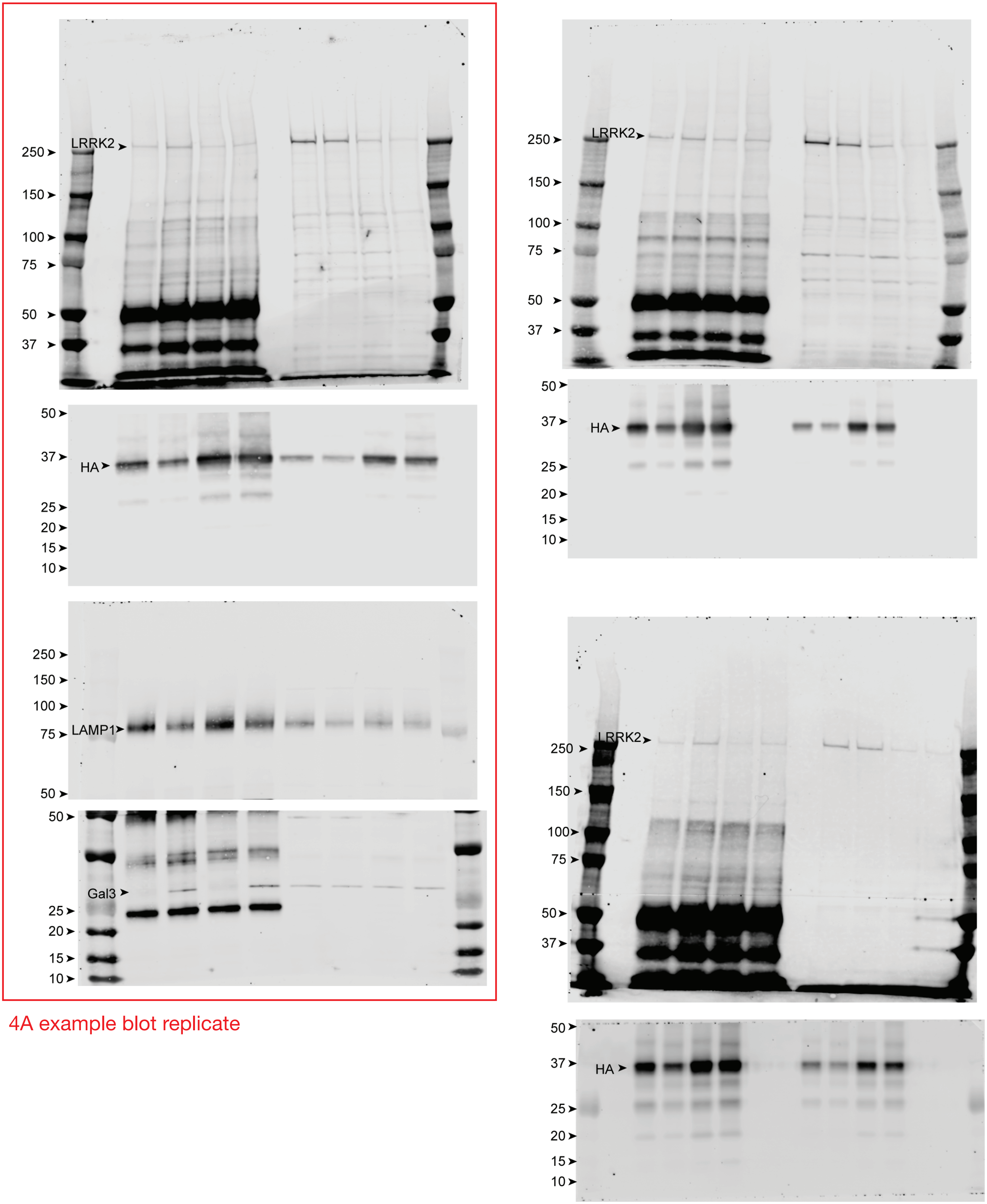

4A example blot replicate

Figure 4A

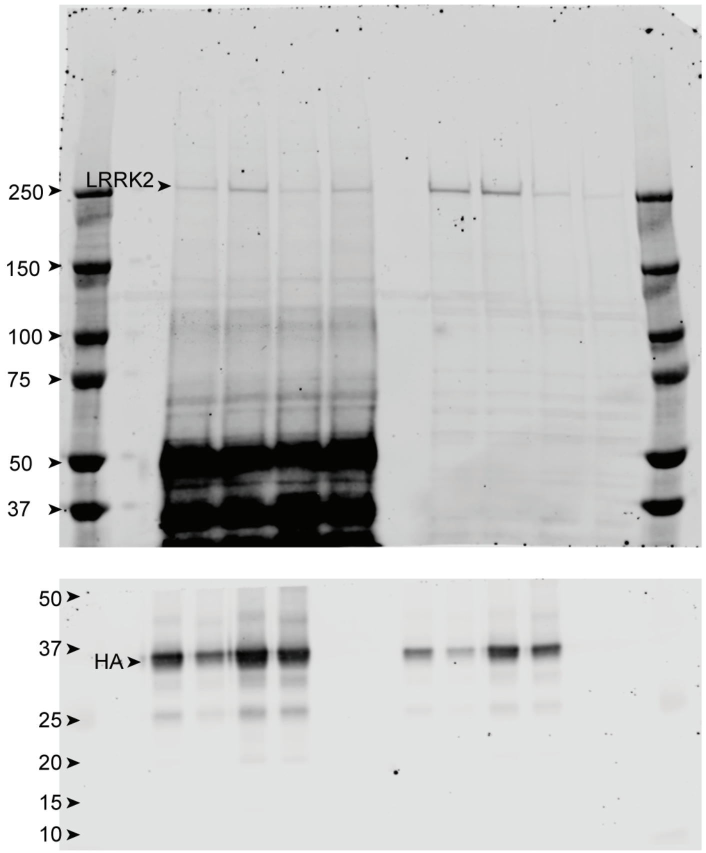

Figure 4B

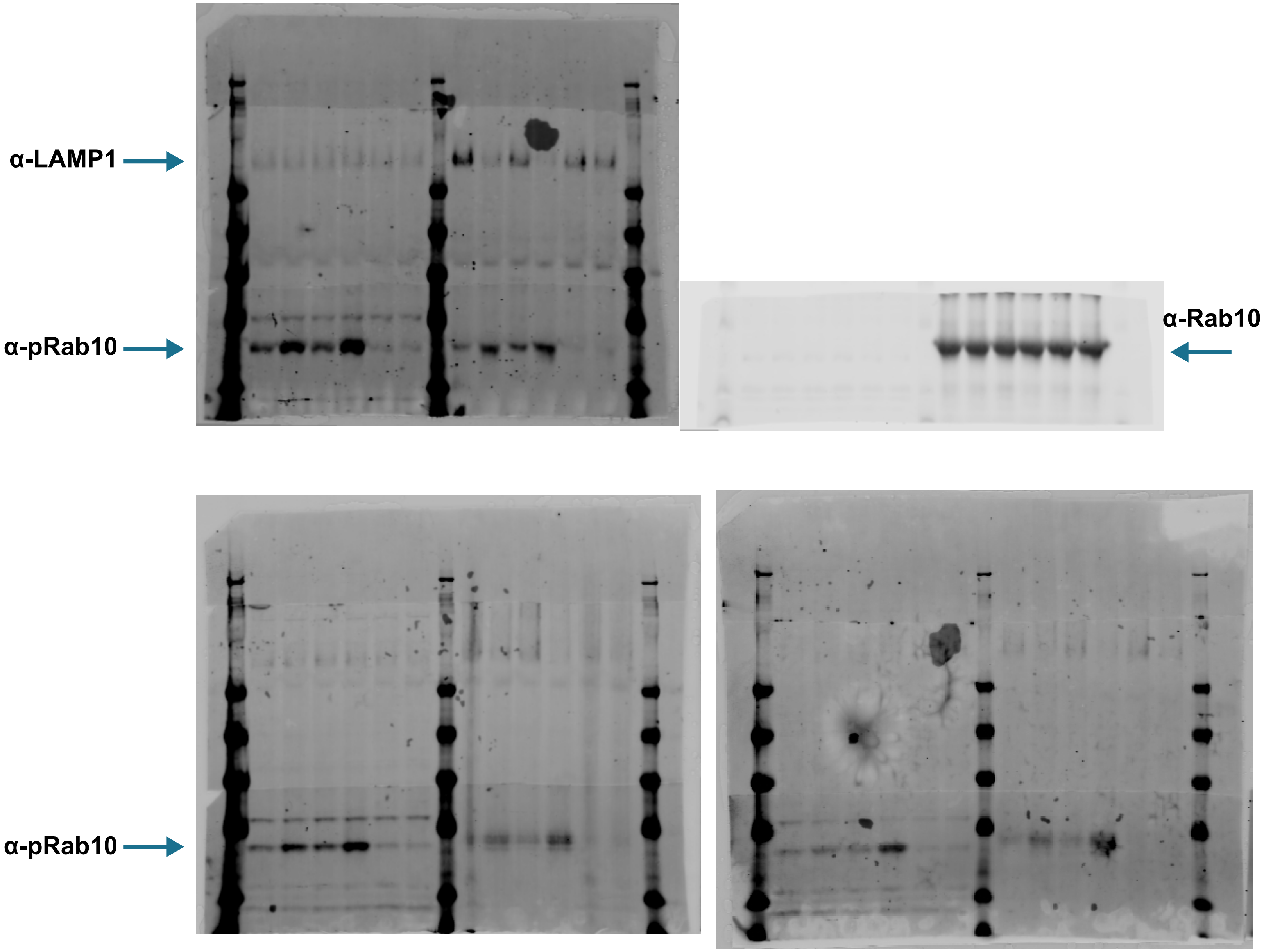

Figure 4C

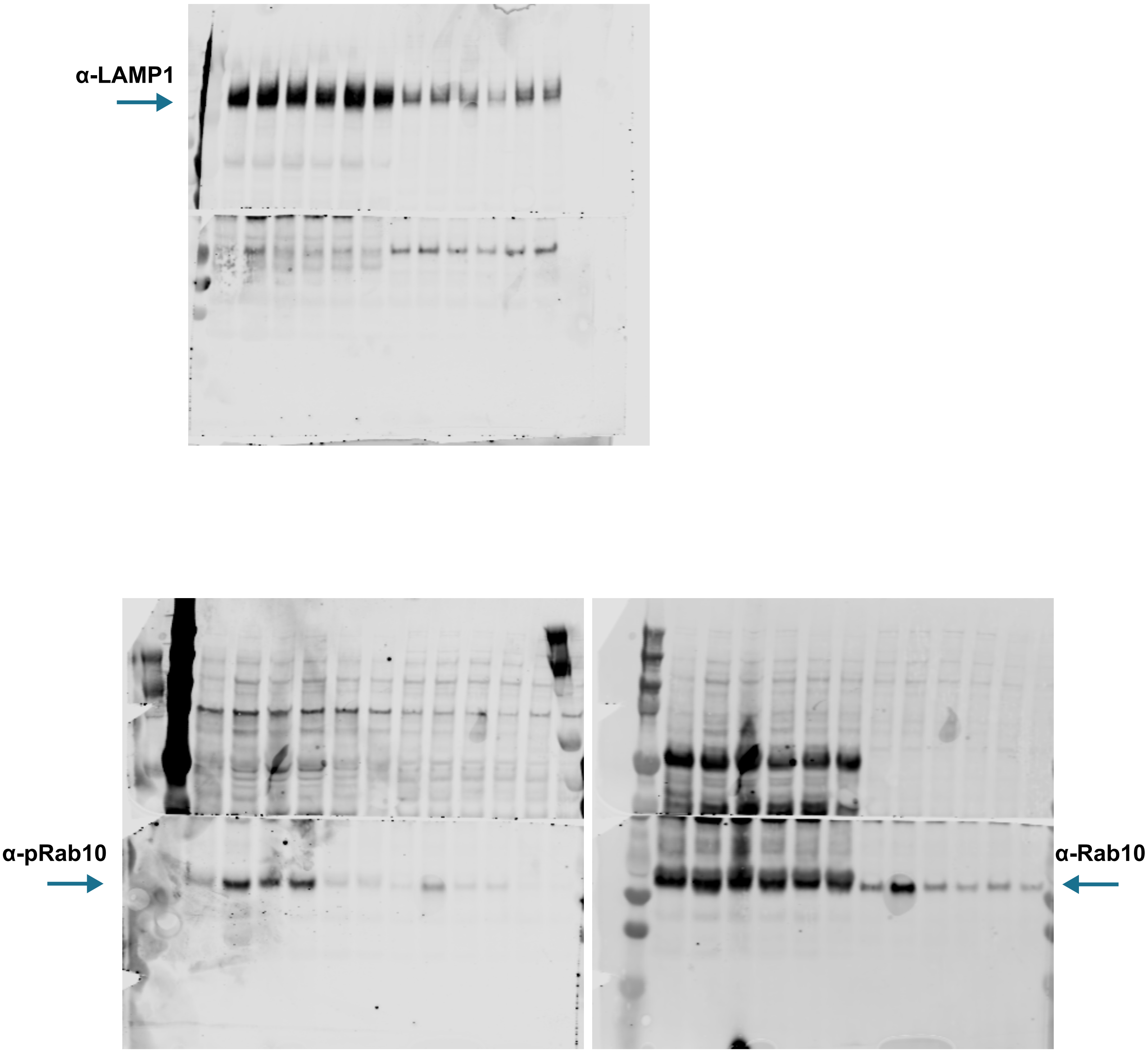

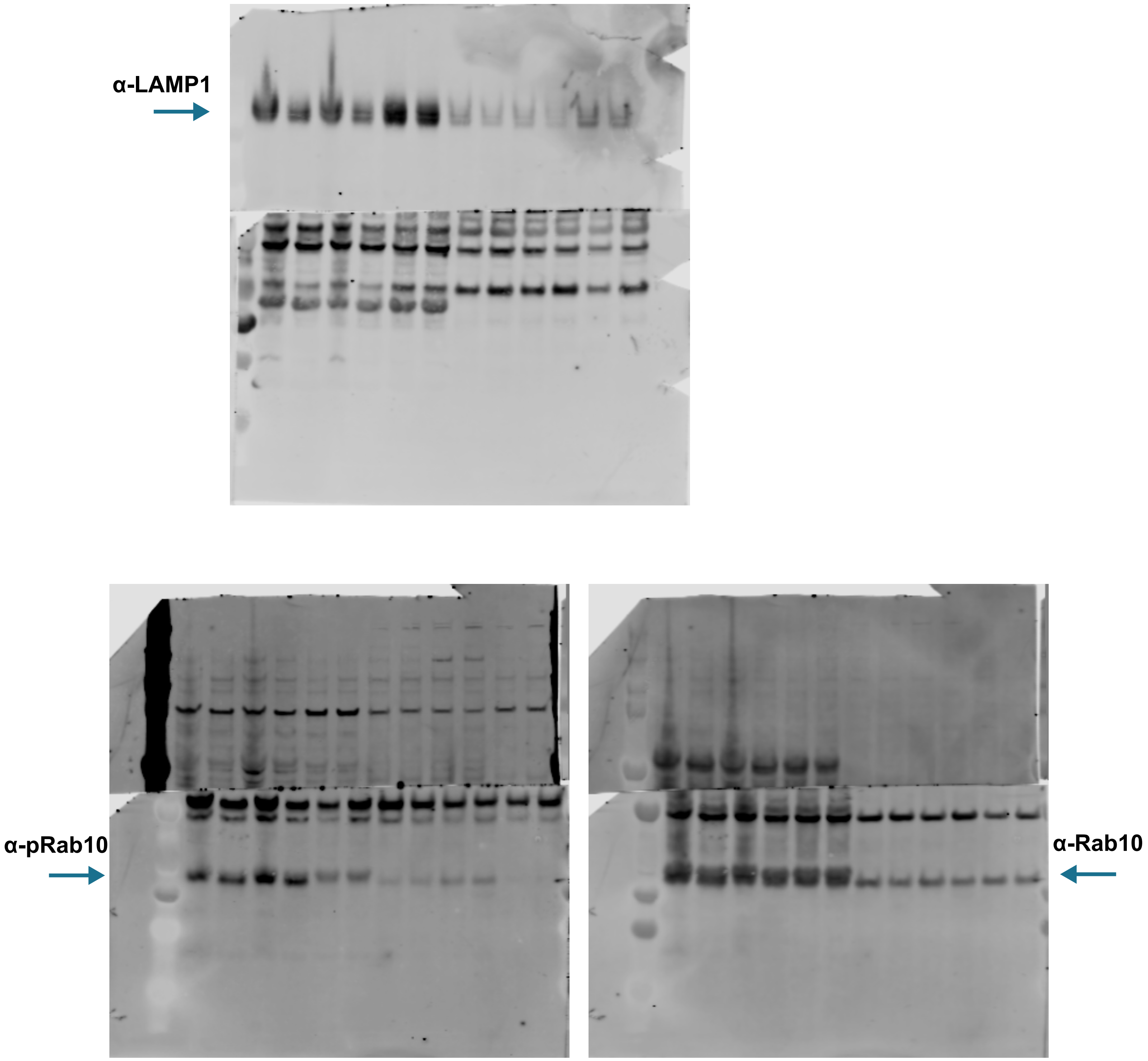
α-LAMP1
α-pRab10
α-Rab10

α-LAMP1

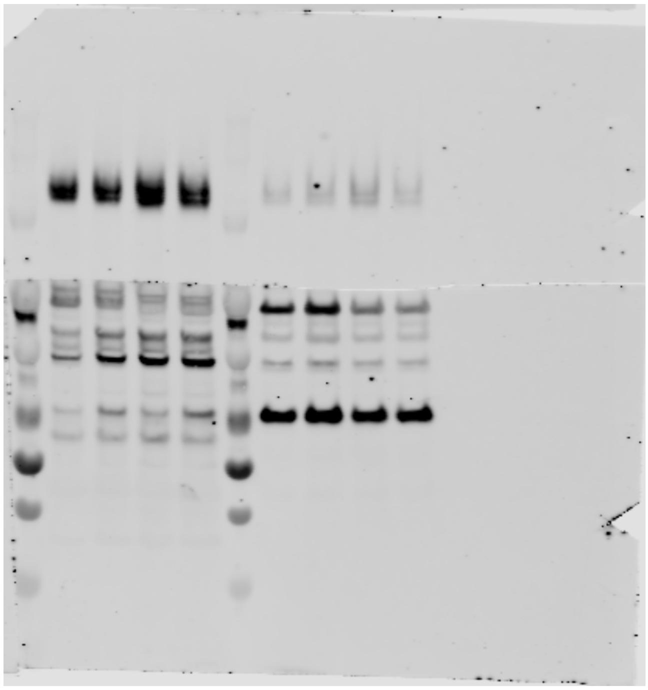

α-pRab10

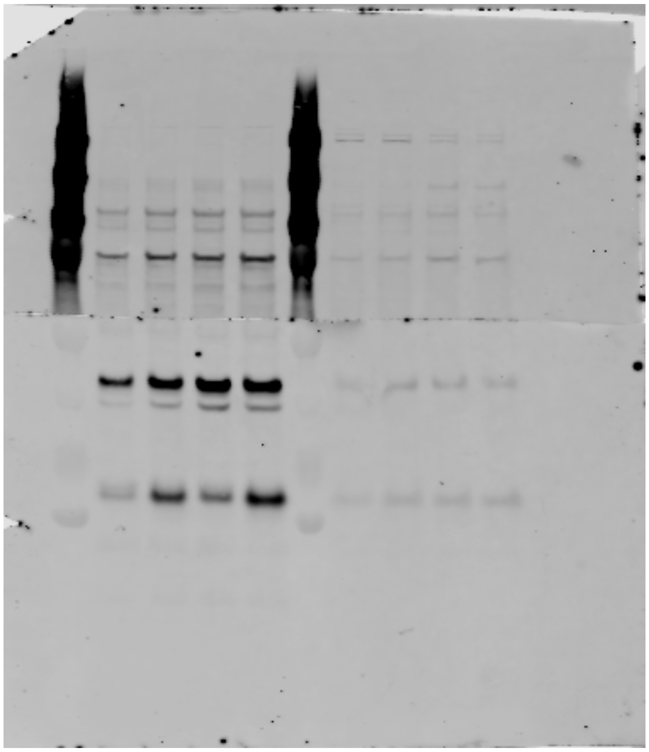

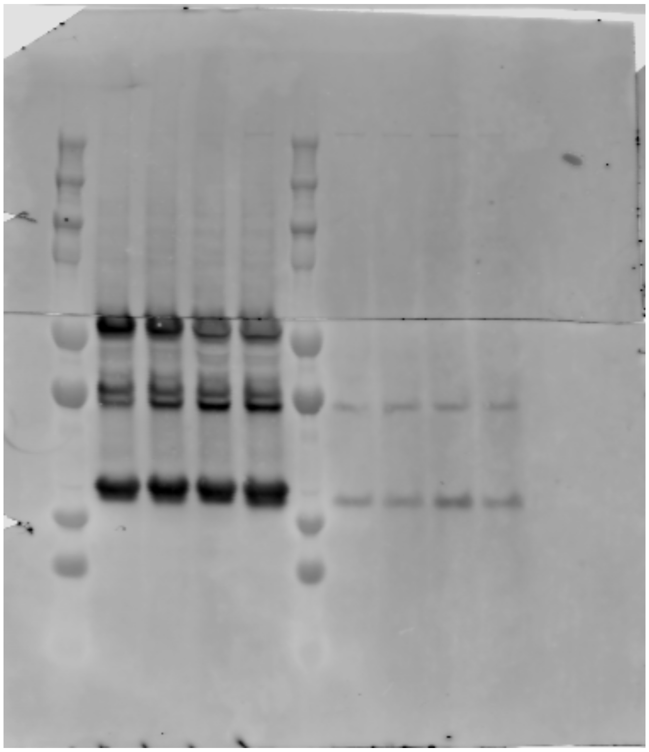

α-Rab10

α-LAMP1

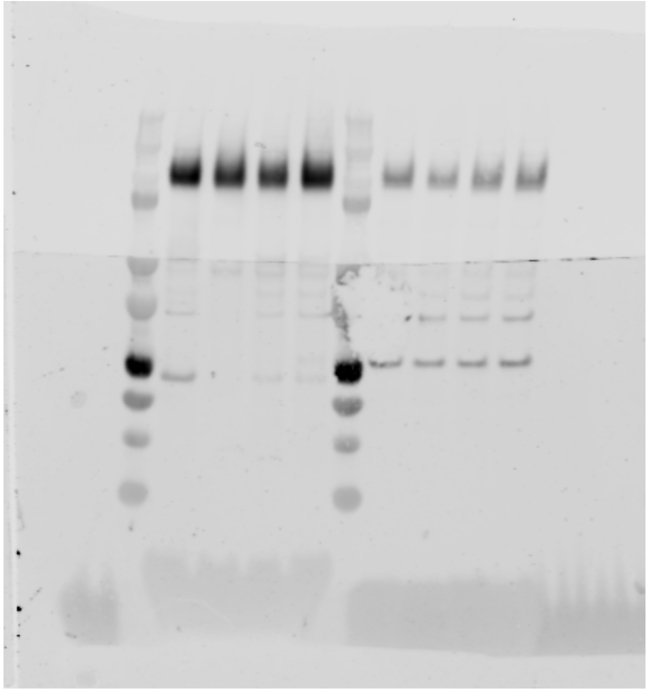

α-pRab10

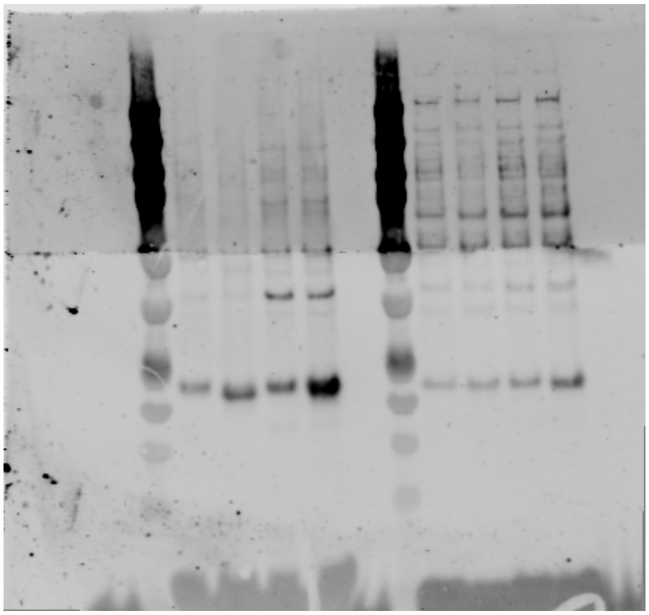

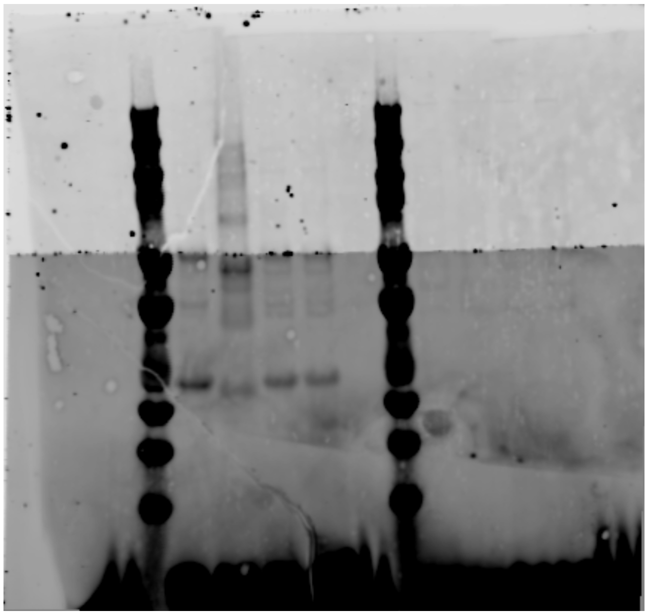

α-Rab10

α-LAMP1

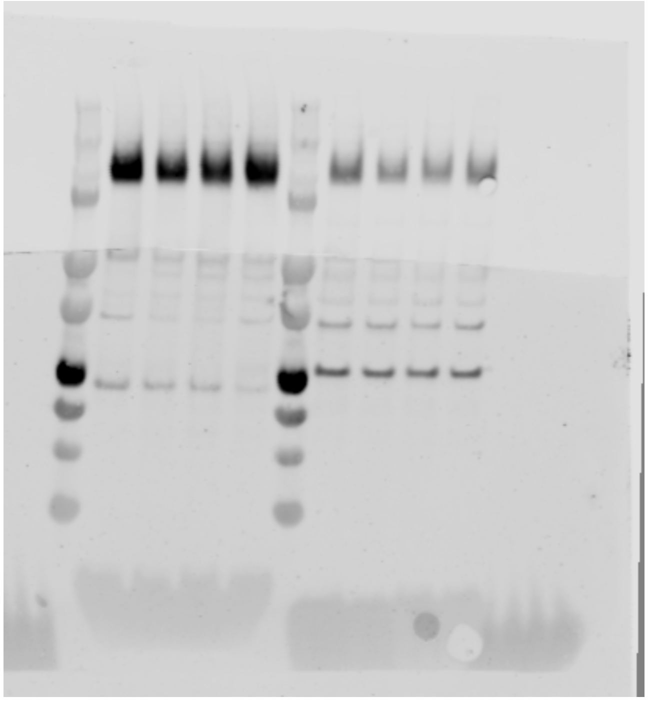

α-pRab10

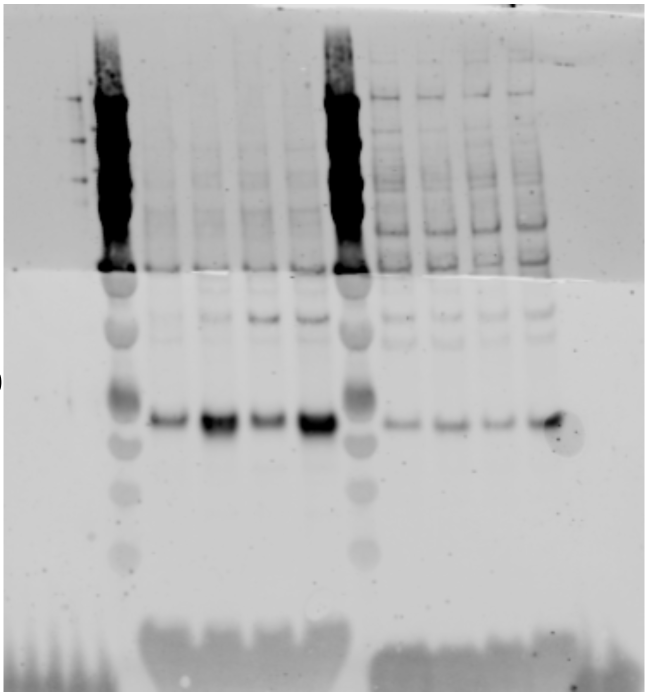

α-Rab10

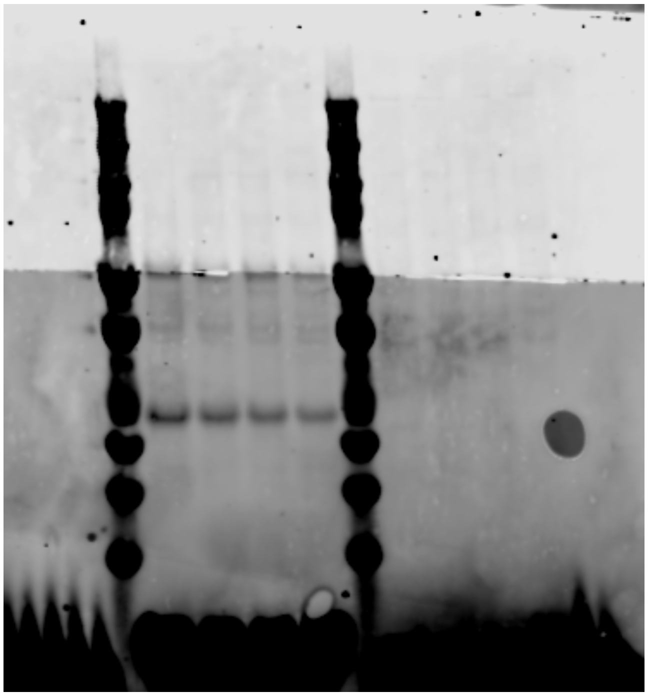

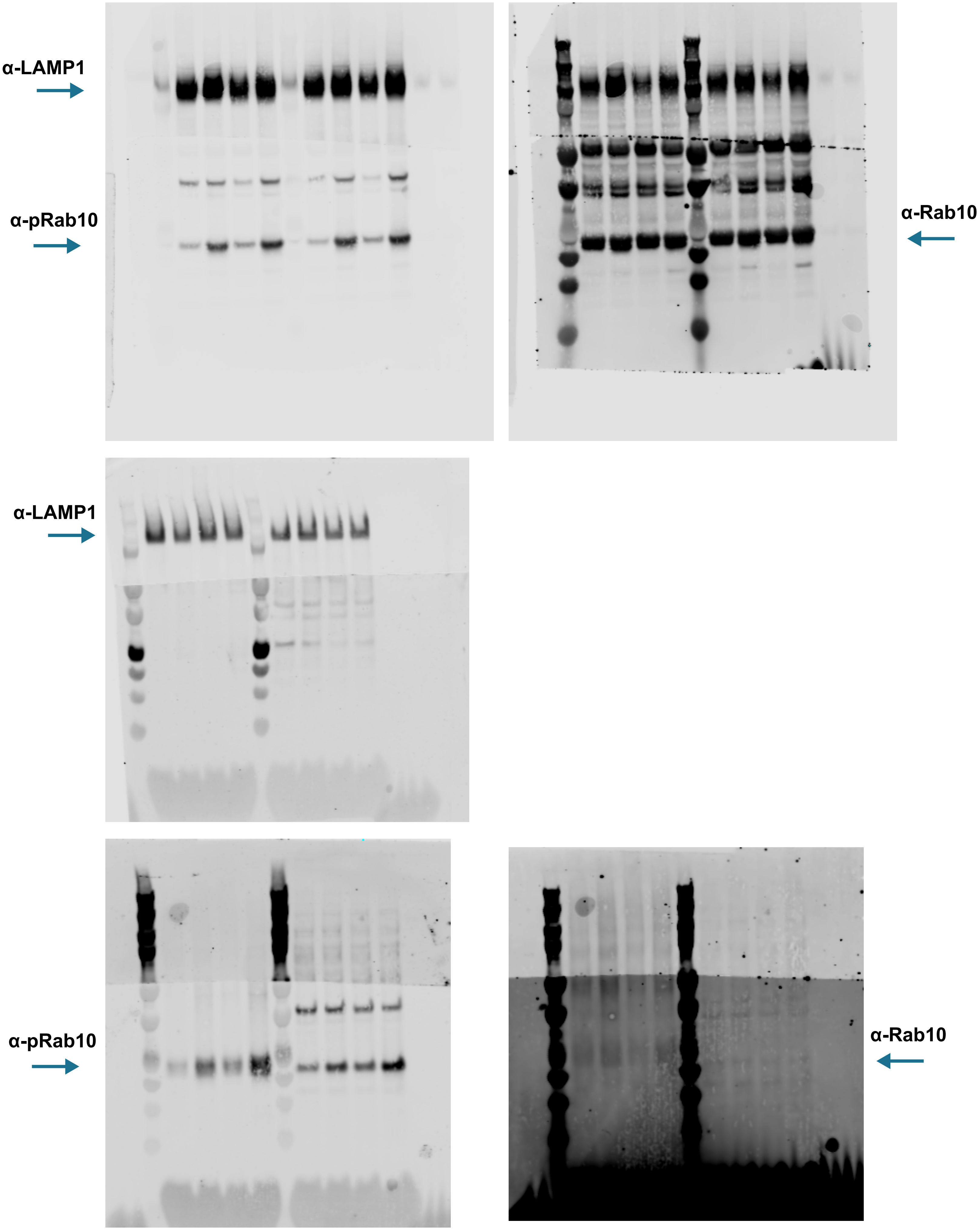
α-LAMP1
α-pRab10
α-Rab10
α-LAMP1
α-pRab10
α-Rab10

Figure 4D

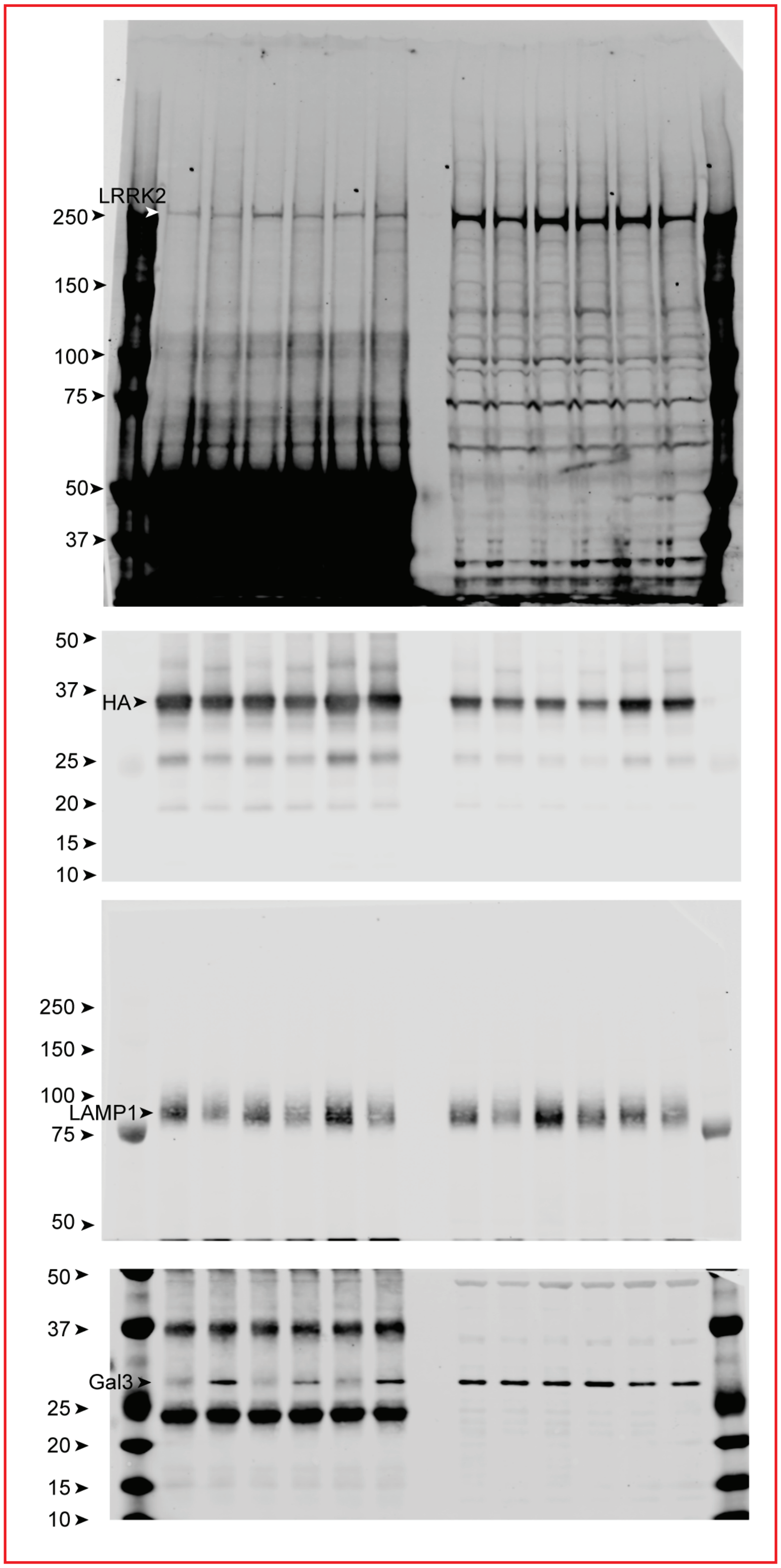

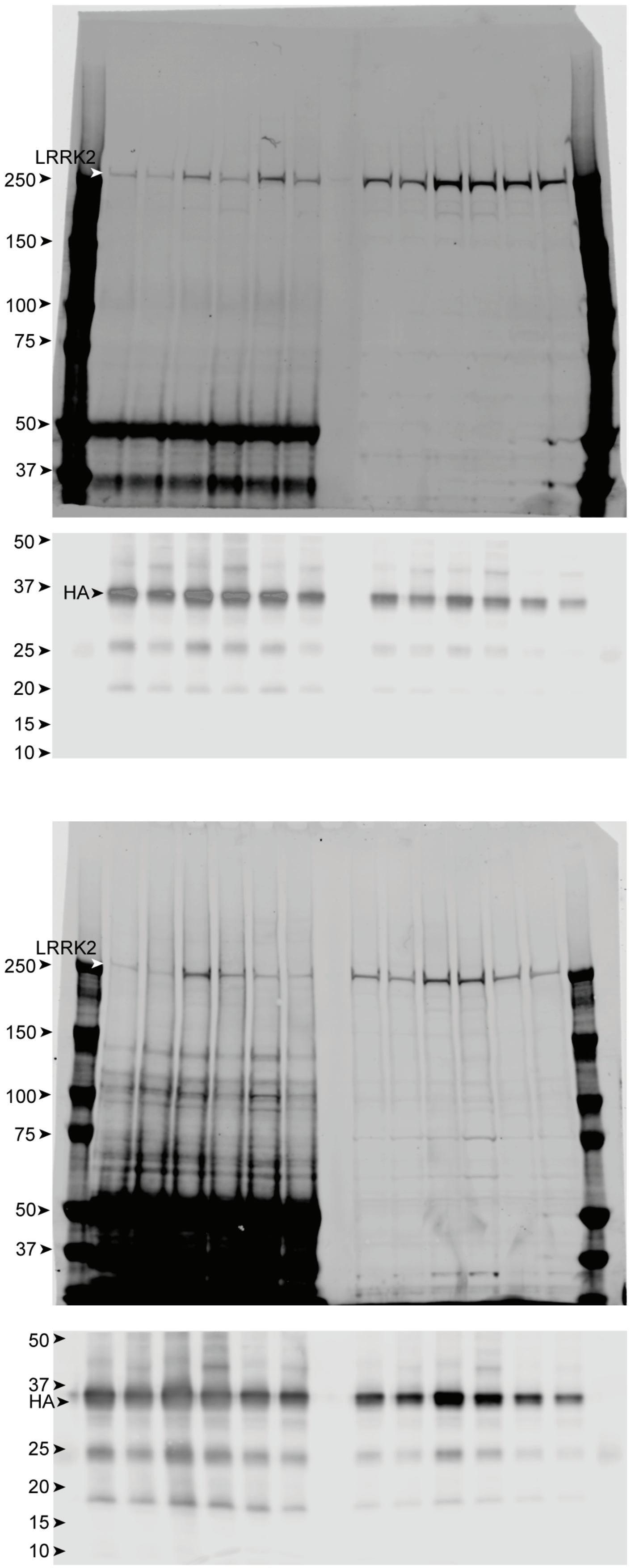

4D example blot replicate

Figure 4D

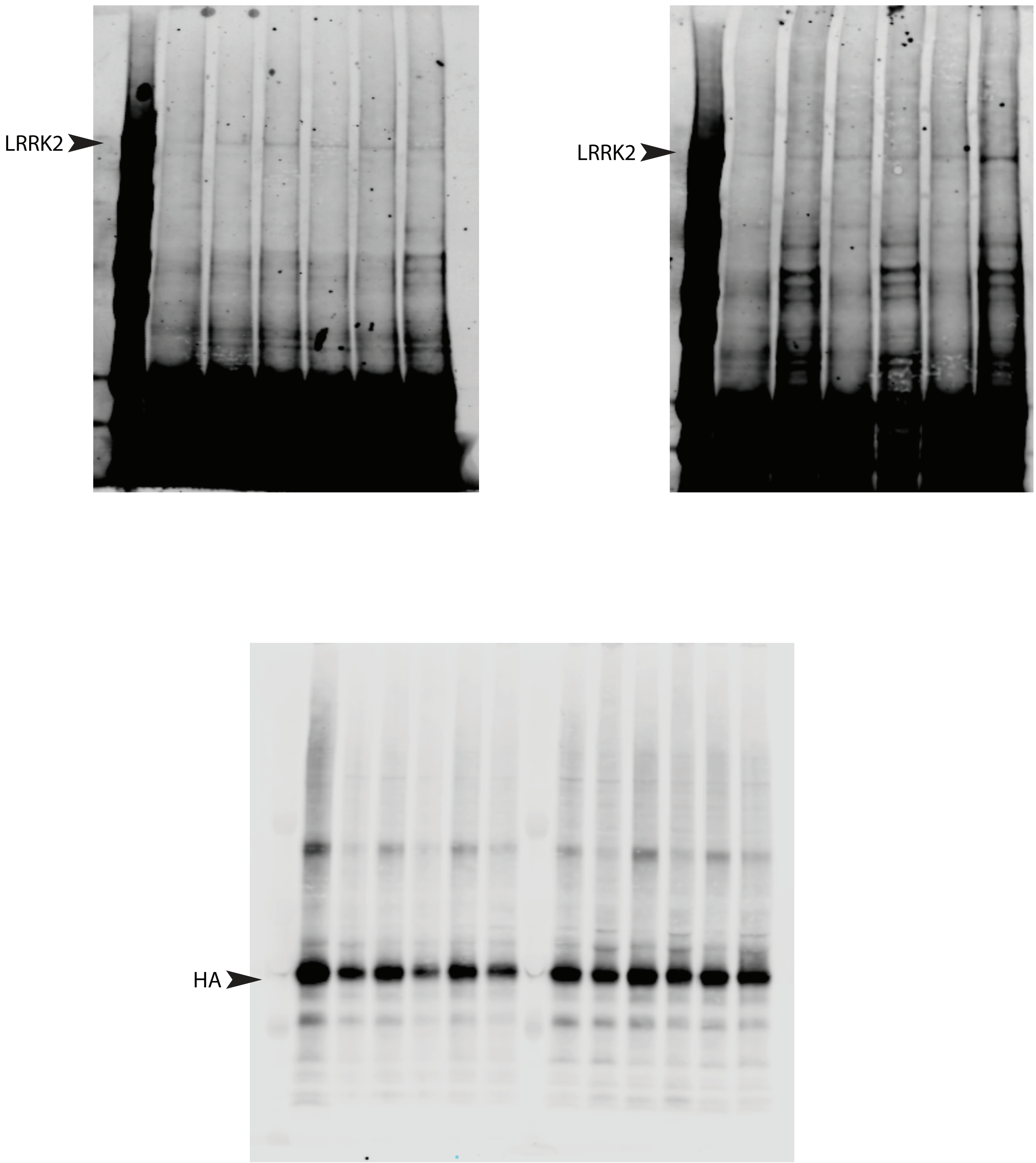

Figure 4D

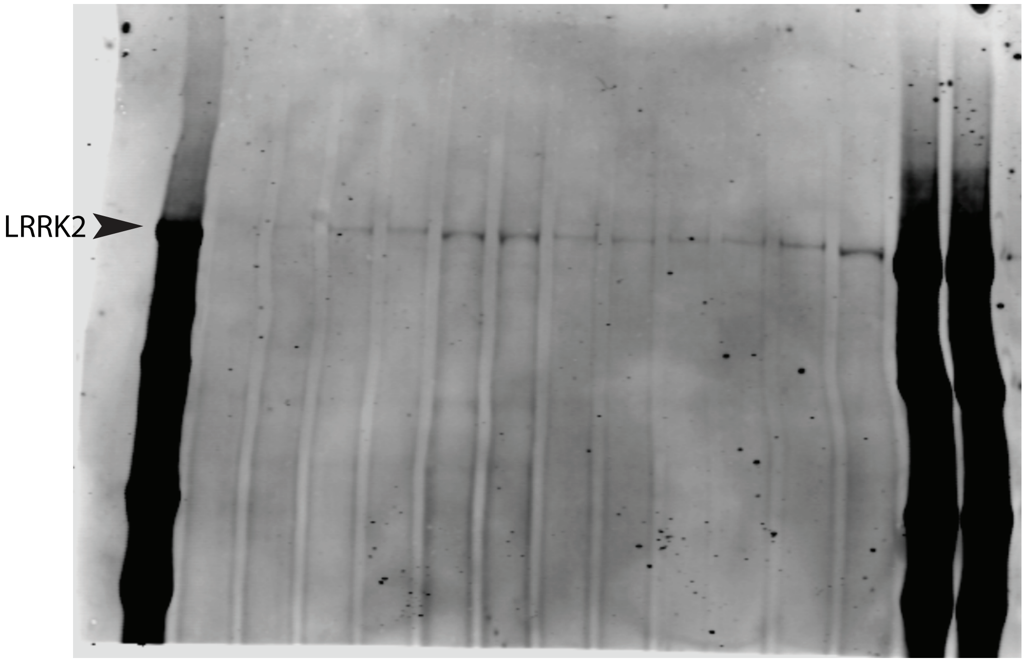

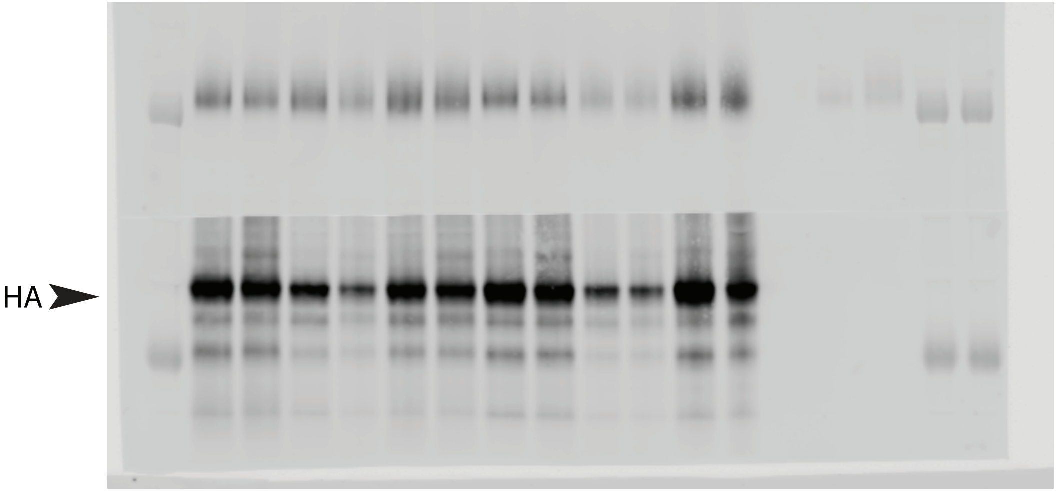

Supplement: Figure 4—source data 2. [file elife-87255-fig4-data2.pdf]
